# The Interplay of Viral and Host Factors in Chikungunya Virus Infection: Targets for Antiviral Strategies

**DOI:** 10.3390/v10060294

**Published:** 2018-05-30

**Authors:** Kai Zhi Wong, Justin Jang Hann Chu

**Affiliations:** 1 Laboratory of Molecular RNA Virology & Antiviral Strategies, Department of Microbiology & Immunology, Yong Loo Lin School of Medicine, National University Health System, 5 Science Drive 2, National University of Singapore, Singapore 117597, Singapore; E0079517@U.NUS.EDU; 2 Institute of Molecular & Cell Biology, Agency for Science, Technology & Research (A*STAR), 61 Biopolis Drive, Proteos #06-05, Singapore 138673, Singapore

**Keywords:** chikungunya virus, host factors, potential therapeutics, interactions, antiviral, viral structural proteins, viral non-structural proteins

## Abstract

Chikungunya virus (CHIKV) has re-emerged as one of the many medically important arboviruses that have spread rampantly across the world in the past decade. Infected patients come down with acute fever and rashes, and a portion of them suffer from both acute and chronic arthralgia. Currently, there are no targeted therapeutics against this debilitating virus. One approach to develop potential therapeutics is by understanding the viral-host interactions. However, to date, there has been limited research undertaken in this area. In this review, we attempt to briefly describe and update the functions of the different CHIKV proteins and their respective interacting host partners. In addition, we also survey the literature for other reported host factors and pathways involved during CHIKV infection. There is a pressing need for an in-depth understanding of the interaction between the host environment and CHIKV in order to generate potential therapeutics.

## 1. Introduction

In recent years, chikungunya virus (CHIKV) has re-emerged as one of the many arthropod-borne viruses (arboviruses) that can pose serious international public health threats [1,2]. CHIKV is an Alphavirus that belongs to the *Togaviridae* family and is transmitted mainly by two species of mosquitoes, namely, *Aedes albopictus* and *Aedes aegypti* [3]. Chikungunya comes from a Makonde word that refers to the bent-up posture that the disease induces [4]. CHIKV can be classified into three different lineages with distinct genotypes corresponding to their respective geographical origins. They include the Asian, East-Central-South African, and West African genotypes [5,6,7,8]. One unique feature, which distinguishes CHIKV from its arguably well-conserved alphavirus cousins, is its high serum viral loads, which can exceed 10^9^ virus particles/mL [9]. This remarkable feature allows easy transmission of the CHIKV to any feeding mosquitos.

According to historical records, CHIKV is likely to have been present since 1779 [10]. However, due to the similar clinical manifestations between CHIKV and dengue infections, CHIKV-infected patients were likely to have been initially misdiagnosed as having dengue infection [10,11]. CHIKV was first isolated and identified during an outbreak in Tanzania in 1953 [4]. Thereafter, many epidemics and outbreaks were documented in a number of African countries in 1958 with thousands of people being infected [12]. Soon after, cases of CHIKV infection mushroomed in many Southeast Asian countries from the 1960s [13]. Between 2005 and 2006, a major unprecedented epidemic of CHIKV swept across many countries within the Indian Ocean territories, where CHIKV was not endemic [13]. During that outbreak, one-third of the 785,000 residents of La Réunion were infected with CHIKV, with a 0.1% fatality rate [14,15]. This ever-expanding geographical range of CHIKV infection seemed unstoppable [16,17]. In 2007, autochthonous CHIKV cases were reported in Italy, making the first instance of an outbreak in temperate regions [18]. Outbreaks and other autochthonous transmission events were subsequently reported in many non-endemic regions like Singapore from 2006 to 2015; France (West-French indies, Caribbean islands) from 2013 to 2015; Spain and Senegal from 2014 to 2015; Argentina, The United States of America, and Kenya in 2016; and Italy in 2010, 2014, and 2017 [19,20,21,22,23,24].

CHIKV-infected patients experience a sudden onset of fever of about 40 °C, together with the trademark symptoms of intense muscle pain in the arms, calves, and thighs, as well as arthralgia in the ankles, elbows, knees, and wrists within 2 to 12 days of infection [25]. This is due to CHIKV’s ability to infect both the skeletal muscle progenitor cells and fibroblasts in the connective tissues of muscles and joints, where a high density of nociceptive nerve endings reside [26,27,28]. In addition, patients also suffer from maculopapular rash, nausea, vomiting, headaches, lymphopenia, and moderate thrombocytopenia [29,30]. The more severe cases, though rare, involve the manifestation of neurological complications. In addition, there are a few cases of CHIKV causing miscarriages and neonatal complications like neonatal encephalopathy after maternal-to-fetal transmission [31,32,33,34]. The immunopathogenesis of CHIKV infection has been extensively reviewed by Burt and colleagues [35].

These acute symptoms usually resolve within two weeks. However, a significant portion of patients experience persistent and/or recurrent joint pains for months or years after contracting CHIKV [36,37]. The mechanism of the progression of the CHIKV disease to the chronic phase remains poorly characterized. However, recent studies have shown that macrophages may play a role in the chronic manifestations of CHIKV [38,39].

Despite the significant healthcare threat posed by the CHIKV, there are still no available vaccines or therapeutics for CHIKV infections [1,35]. Patients are usually given analgesics and anti-inflammatory drugs to relieve symptoms. Even though there are a number of anti-CHIKV compounds being reported, precise mechanistic data of these compounds, as well as efficacy studies in mouse models, are lacking. Ribavirin and chloroquine are the only two drugs that have been tested in clinical trials [40]. Despite having promising in vitro data, chloroquine was found to be ineffective in clinical trials [41,42]. On the other hand, Ribavirin was found to be effective in alleviating chronic symptoms. However, the clinical trial cohort (20 patients) was too small to provide conclusive evidence [43]. Therefore, there is a pressing need to identify and develop novel antivirals to combat CHIKV infection.

Although attempts to develop drugs that specifically target viral proteins have proven to be successful, the process is rather time-consuming and costly [44,45,46,47,48]. Furthermore, these compounds were found to often display narrow spectrum activities [49]. One promising approach is to target host factors that are known to be hijacked by viruses using either new compounds or to repurpose existing approved drugs [50,51,52]. However, there are still many gaps in the current knowledge of basic virology and replication of CHIKV, which poses difficulties for the discovery of anti-CHIKV therapeutics. The purpose of this review is to provide an overview of the existing research thus far on the interplay of viral and host factors during CHIKV infection. We hope this will guide further research into potential druggable targets in the discovery of potential therapeutics.

## 2. CHIKV and Its Replication Cycle

CHIKV is an enveloped, spherical virus with a diameter of about 60–70 nm [53,54,55]. The CHIKV genome consists of a single-stranded, positive-sense, linear RNA that is about 11,800 nucleotides long [56,57]. The 5′ end of the positive-sense RNA genome possesses a 7-methylguanosine cap, and the 3′ end has a polyadenylated tail [58]. There are two open reading frames (ORF) found in the CHIKV genome, one for the non-structural proteins, the other for the structural proteins. The first ORF encoding the non-structural proteins (nsP1, 2, 3, and 4) makes up nearly two-thirds of the genome [56]. The remaining one-third portion encodes for the five structural proteins (capsid, E3, E2, 6K, and E1) [56]. The CHIKV genome contains three non-translated regions (NTR) [56,58]. The 5′ NTR consists of 76 nucleotides, while the 3′ NTR is composed of 526 nucleotides [59]. The remaining NTR, which is 68 nucleotides long, is found between the two ORF and carries a promoter sequence that allows the generation of the 26S subgenomic RNA that encodes all the structural proteins [59]. The CHIKV genome is enclosed within the nucleocapsid core in the mature virion. The nucleocapsid, made up of 240 units of capsid proteins [56], is in turn surrounded by an envelope made up of a host-derived lipid bilayer. The CHIKV envelope is studded with 80 sets of trimeric spikes, with each spike containing three E1-E2 heterodimers, which mediate entry into host cells [60,61].

Upon binding to the host cell receptor via E2 protein, the CHIKV particle is endocytosed by the host cell via the clathrin-mediated pathway in a process involving Eps15 (epidermal growth factor receptor pathway substrate 15), although clathrin-independent entry has also been reported [62,63,64,65,66]. Within the early acidic endosome, the low pH environment initiates the fusion of the viral envelope and the endosomal membrane in a process mediated by the E1 protein [63,65,66,67,68]. The membrane fusion process occurs rapidly (within 40 s after endocytosed) in Ras-related protein 5 (Rab5)-positive endosomes and is found to be highly dependent on the presence of cholesterol [66]. Thereafter, the virus disassembles releasing the viral RNA genome into the cytosol [68,69]. By hijacking the host translational machinery, the first two-thirds of the viral RNA is rapidly translated into a polyprotein (P1234), which consists of all the four non-structural proteins [53,56]. However, this polyprotein (P1234) makes up to only 10% of all the translated transcripts due to the presence of an opal stop codon between the nsP3 and nsP4 gene [58]. The remaining 90% is made up of the P123 polyprotein. The opal stop codon is believed to play a role in regulating the levels of the nsP4 by the alphaviruses [70]. P1234 is subsequently cleaved in *cis* by the viral protease nsP2 yielding P123 and nsP4 [53]. This allows the formation of an initial but unstable replication complex, which allows the synthesis of negative-sense strand viral RNA. At this phase of the replication cycle, structures known as spherules, which are derived from the plasma membranes, can be observed to be studded along the plasma membrane. The unstable P123-nsP4 complexes are proposed to be localized near the neck of these spherules, which functions in protecting the double stranded RNA intermediates from detection and degradation [71,72,73].

As the infection progresses, the spherules become internalized and contribute to the formation of a membranous structure called virus-induced type 1 cytopathic vacuoles (CPV-I) [72,73,74]. A recent study by Thaa and colleagues showed that most of the spherules induced by CHIKV infection remain at the plasma membrane, and that the internalization of the spherules is dependent upon the activation of the phosphatidylinositol-3-kinase-Akt-mTOR pathway [75]. The CPV is a typical membranous structure (derived from both endosomes and lysosomes) found in *Alphavirus*-infected cells that allows active synthesis of both the negative-sense RNA intermediate and the positive-sense viral RNA [72,73,74]. The accumulation of the P123-nsP4 complexes eventually crosses a certain stoichiometric threshold concentration leading to the cleavage of P123 in *trans*, releasing nsP1 protein [76,77]. This results in a more stable replication complex within CPV-I, made up of nsP1, P23, and nsP4 [78,79,80]. A stable replication complex is subsequently formed upon the final cleavage of P23 into nsP2 and nsP3 proteins. This induces a switch from the synthesis of the negative-sense RNA intermediate to the synthesis of both the full-length viral genome and the 26S subgenomic RNA [74,81].

The 26S subgenomic RNA (which encodes only the structural proteins) is then translated into structural polyproteins [56]. Once the full length capsid protein has been translated, it undergoes autocleavage almost immediately, while the translation of the remaining structural proteins continues [57,82,83,84]. In addition, upon its self-cleavage, the capsid protein is able to recognize the full-length genomic viral RNA [82,83,84]. The capsid proteins then oligomerize, packaging the viral genome into the nucleocapsid core [85,86]. Again, similar to the non-structural proteins, two types of structural polyprotein products can be identified after the self-cleavage of the capsid protein. The presence of the major structural polyprotein (consisting of E3, E2, 6K, and E1) and the minor one (10–18%) (containing only E3, E2, and TF) have been attributed to the slippery codon motif (UUUUUUA) found on the 6k gene, resulting in a −1 ribosomal frameshifting event [87,88]. These structural polyproteins are then directed by the signal peptide found on the N-terminus of the E3 protein to the endoplasmic reticulum membrane, where they undergo complete cleavage into individual proteins (pE2 (E3-E2), 6K or TF, and E1) by host proteases [89]. E1 and pE2 proteins associate noncovalently, forming a heterodimer complex, which undergoes posttranslational modifications as it gets shuttled via the Golgi secretory pathway. The host furin cleaves pE2, resulting in a mature E2 viral protein [90,91,92,93,94]. Soon after, fully developed nucleocapsid cores carrying full-length, positive sense genomes are recruited to the cell plasma membrane where they bud out of the host cells, simultaneously acquiring a portion of the host plasma membrane studded with mature envelope glycoproteins, making up the envelope of the mature virion [53].

It is also interesting to note that in the late phase of infection, another type of cytopathic vacuole called CPV-II can be observed in infected cells [95,96,97]. In contrast to CPV-I, CPV-II originate from the trans-Glogi network [98,99]. Within these vacuoles, the viral envelope glycoproteins (E1 and E2) have been found to be arranged in a hexagonal lattice and packed in arrays of helical tube-like structures [98,100]. In addition, nucleocapids have also been observed along the periphery of the cytoplasmic side of CPV-II [96,98,100]. Given that these structures are in close proximity to the plasma membrane, it is postulated that they aid in viral assembly and/or transport of the envelope proteins to the plasma membrane and viral release via a second mechanism, exocytosis [98,101].

## 3. Interplay of Host Factors with CHIKV Structural Proteins

Structural proteins are known to be involved in processes like entry, fusion, uncoating of virus particle, assembly of virions, and budding. Here, we present a brief review of the functions of each individual viral proteins and their reported interacting host factors.

### 3.1. Capsid Protein

The CHIKV capsid protein is a compact multifunctional protein of 261 amino acids with a molecular weight of about 30 kDa [58,102,103]. Unlike the New World encephalitic alphaviruses, the capsid proteins of CHIKV (an Old World arthritogenic alphavirus) do not seem to be involved in the shutting down of the host transcriptional processes [104]. Instead, the Old World arthritogenic viruses rely on nsP2 for inducing host transcriptional and translational shutoff [104].

The capsid protein is made up of three main regions (regions 1, 2, and 3) [105,106]. Region 1 (1–80 aa) being highly basic in nature (Arg-, Lys-, and Pro-rich), is proposed to be able to bind RNA in a non-specific manner and may also be involved in protein interactions that inhibit host transcription [106]. In contrast, region 2 (81–113 aa) binds specifically to the full length viral RNA genome and also plays an important role in the oligomerisation of other capsid proteins in order to form mature nucleocapsid particles [85,106,107,108,109]. Region 3 is a serine protease containing a conserved catalytic triad (His 139, Asp 161, and Ser 213) that is able to cleave itself in *cis* and inactivate itself by binding its active site with its C-terminal tryptophan residue [53,82,83,110]. A recent study reported that the CHIKV capsid is also able to exhibit *trans*-cleavage properties [111]. In addition, the hydrophobic pocket (containing interacting residues: Val 130, Gly 131, Val 134, Met 135, Trp 245, and Val 250) located on region 3 was found to interact with Pro 404 of the E2 cytoplasmic domain, which is believed to occur during mature particle assembly [102,106,112]. Moreover, dimerization of two capsid protein monomers relies on the interaction of the Tyr 186 residue from one monomer with two Asn residues at positions 188 and 220 from the other monomer, all of which are located on region 3 [102].

The CHIKV capsid protein has been reported to possess both one nuclear import (NLS) and two nuclear export signals (NES) (44–53 aa & 143–155 aa), which allows the protein to traverse actively between the nucleus and cytoplasm [113,114]. In addition, two host proteins, karyopherin α (Karα) and chromosomal maintenance 1 (CRM1), have been found to be involved in the active nuclear import and export of the CHIKV capsid protein, respectively [113]. Interestingly, both NES are required to be intact for CHIKV capsid to be exported. Mutation of the NES near the N-terminus (44–53 aa), by replacing the Lys 51 and Met 53 with alanines, resulted in the retention of the CHIKV capsid within the nucleus [114]. Unexpectedly, this lead to the blockage of the host nuclear import system through a mechanism which remains unknown [114]. In addition, Taylor and colleagues showed that mutating the nucleolar localization sequence (NoLs) within the N-terminus results in an attenuated phenotype with smaller plaques and reduced virulence in mice while still being able to elicit an immune response [113,114,115]. The precise location of the NLS of the CHIKV capsid has yet to be confirmed. Jacobs and colleagues suggested that the location falls between 1 and 83 aa of the capsid protein, whereas Thomas and colleagues reported that it should be between 60 and 99 aa.

Given that the capsid protein is such a crucial, multifunctional viral protein with such an array of functions, more efforts could be channeled to uncover the possible interactions with other potential host factors.

### 3.2. E3 Protein

E3 proteins carry a signal peptide (a series of polar residues) at their N-terminus, which is crucial for targeting the structural polyprotein towards the endoplasmic reticulum for initial processing [53,106]. Despite being only 64 amino acids long (~7.4 kDa), it is necessary for the stabilization and maturation of the E2 glycoprotein [58,87,116,117]. From crystal structures, the E3 protein was observed to bind exclusively to the E2 protein [118]. It requires the host furin enzyme to mediate the maturation of the E2 protein by cleaving it in the trans-Golgi system only after dimerization with other available E1 glycoproteins is complete [106,118]. After cleavage, the E3 protein continues to associate non-covalently with the E1-E2 spikes by relying on the interaction between the Tyr 47 residue on E3 and the Tyr 48 on the E2 [119]. It is not incorporated into the virus and will dissociate when the entire complex is exposed to neutral pH at the plasma membrane surface [119]. Upon dissociation, the acid-sensitive region between the E2 and E1 glycoproteins gets exposed, priming the E1 protein for activation upon contact with low pH during entry [106,119]. E3 therefore plays an important role in protecting the envelope glycoproteins from low pH and preventing their premature activation. No other host factors that interact with the E3 protein have been reported.

### 3.3. E2 Protein

The E2 protein (423 aa, ~40 kDa), a type I transmembrane glycoprotein, has long been known to be the main antigenic and receptor binding protein for CHIKV [58,120]. The CHIKV exhibits a wide tropism by being able to replicate in many invertebrate and vertebrate cells [27,65,121,122,123]. In addition, E2 proteins also serve as stabilizing factors (together with E3 proteins) for the E1-E2 heterodimer during the entire intracellular transport through the secretory pathway, where folding and post-translational modifications take place, and finally to the plasma membrane [106].

To date, no bona fide receptor has been identified for CHIKV. However, there are host factors that have been reported to aid in viral entry. For instance, prohibitin (PHB) was identified to aid in the binding of CHIKV to CHME-5 microglial cells [124]. Wintachai and colleagues also suggested that the possibility of additional co factors that assist the CHIKV entry as poor infection was observed in U937 monocytic cells that also express PHB [124]. In a follow up study, Wintachai and colleagues showed that flavaglines (prohibitin ligand) was able to inhibit CHIKV entry by preventing the CHIKV and prohibitin from colocalising in HEK293T/17 cells [125,126]. These studies suggest that PHB may be the putative receptor for CHIKV. In addition, ATP synthase β subunit was found to be involved in the entry of CHIKV into *Aedes aegypti* mosquito cell lines [127]. Additional host factors like protein tyrosine phosphatase non- receptor type 2 (PTPN2), fibril-forming collagen (COL1A2), and actin gamma 1 (ACTG1) were also shown to interact with the E2 proteins in immunoprecipitation experiments [128]. However, further mechanistic studies are required to understand the role of these proteins during CHIKV infection.

Jemielity and colleagues showed that overexpression of either human T-cell immunoglobulin and mucin-domain containing proteins 1 (hTIM1) or AXL receptor tyrosine kinase (also known as UFO) of the TAM family of kinases promoted entry of CHIKV by at least 8-fold in HEK293T cells [129]. Their work also showed that CHIKV entry is phosphatidylserine (PS)-dependent, as the PS binding deficient hTIM1 variant did not support viral entry [129]. This suggests that PS is exposed on the membrane of the CHIKV, similar to other enveloped viruses, including Pichinde virus, vesicular stomatitis virus, and vaccinia virus [130,131]. By taking advantage of the exposed PS, the CHIKV is able to enter the cells. However, the authors noted that the blocking of hTIM1 receptors was less effective in preventing CHIKV entry in Huh7 cells [129]. In addition, there are no reported interactions between hTIM1 and the CHIKV receptor-binding protein, E2. This suggests that the PS-recognising TIM and TAM receptors may not be the bona fide receptors for CHIKV but instead may play a role as attachment factors that enhance CHIKV infectivity.

Recent work has shown that only the two surface-exposed domains (domains A and B) of CHIKV E2 are able to bind to cells [132]. Binding of the CHIKV with soluble GAGs was found to be able to inhibit CHIKV infection by up to 90% [132]. In addition, only domain A was able to bind to cell-surface glycosaminoglycans (GAGs)-deficient cells, while domain B was found to interact exclusively with cells expressing GAGs on their cell surface [132]. These results suggest that CHIKV could employ more than one entry mechanism, which most probably explains the wide tropism of cells observed. Therefore, more efforts could be directed to uncover other host factors/potential receptors that could interact with the CHIKV E2 proteins.

### 3.4. 6K/TF Protein

The TF (~8.3 kDa) protein possess the same N terminus as 6 K (61 amino acids ~6.6 kDa) but differs by having a longer basic C terminus (~15 residues) instead of a shorter, hydrophobic C terminus found on 6 K [58,87,88]. Unlike other structural proteins, the exact functions of both 6K and TF have not been clearly elucidated [133]. However, studies on other alphaviruses have suggested that these viral accessory proteins mediate membrane permeability and viral budding, and may also be involved in forming ion channels [87,134,135]. In addition, both 6 K and TF are also incorporated in low levels into mature virions and are crucial in preserving both the stability and infectivity of the virus [136,137,138]. However, no host factor has been found to interact with either of these proteins.

### 3.5. E1 Protein

The E1 protein (435 amino acids, ~44 kDa), a class II viral fusion protein, mediates the fusion of the viral envelope with the host endosomal membrane after endocytosis [58,120,139]. This results in the release of the nucleocapsid into the cytoplasm. A single mutation of Ala 226 to a valine residue on the E1 glycoprotein enhanced the dissemination of CHIKV into the secondary organs of *Aedes albopictus* mosquitos. Moreover, this phenomenon was also detected in the suckling mice [140]. This mutation coincided with the emergence of *Aedes albopictus* as a second transmission vector during the Indian Ocean epidemic in 2004 [15]. Moreover, in a recent study by Hoornweg and colleagues, this mutation reinforced the cholesterol-dependent membrane fusion of the CHIKV with the host endosomal membranes [66]. In another recent paper, another two epistatic mutations (E1:K211E together with E2:V264A) were also found to notably enhance transmission (62 fold), infection (13 fold), and dissemination (15 fold) in *Aedes aegypti* mosquitos [141]. However, the exact mechanisms and possible interacting host factors that may facilitate the enhanced fitness of the virus are still unknown.

In an attempt to uncover host proteins that interact with E1, Dudha and colleagues performed a yeast two-hybrid (Y2H) screening on a human brain cDNA library [128]. The screen was able to identify 5 interacting host proteins (copper metabolism (Murr1) domain containing 1 (COMMD1), thrombospondin 1 (THBS1), dynein, cytoplasmic 1, heavy chain 1 (DYNC1H1), ATPase Na1/K1 transporting beta 3 polypeptide (ATP1B3), and microtubule-associated protein 1B (MAP1B)). Of these, four hits (COMMD1, THBS1, DYNC1H1, and ATP1B3) passed and were validated via immunoprecipitation and ELISA [128]. However, the biological significance and exact mechanisms of the interactions have yet to be explored.

A second study reported that bone marrow stromal antigen 2 (BST-2 or tetherin or CD317) was able to restrict and trap CHIKV on the surface of the host plasma membrane by engaging the E1 glycoprotein [142]. BST-2 knockout mice suggest that BST-2 is able to protect the lymphoid tissues and regulate the inflammatory response induced by the CHIKV. Moreover, another study showed that the longer isoform of BST-2 was found to specifically block the exit of alphaviruses (e.g., SFV and CHIKV) efficiently [143]. In addition, although rubella virus and dengue virus share similar virion structure as the alphaviruses, they responded differently to the presence of BST-2, with the dengue virus not getting inhibited at all [143].

All in all, though much efforts have been focused on uncovering potential interacting host factors, there are still many gaps in our knowledge understanding the interaction of the host factors with the CHIKV structural proteins.

## 4. Interplay of Host Factors with CHIKV Non-Structural Proteins and Functions

The major function of CHIKV non-structural proteins (nsPs) is to replicate the viral genome for translation of structural proteins and for packaging into progeny virions. Aside from this, most nsPs also have additional functions outside of the replication complex. Here, we will present a brief review of the functions of each individual viral protein and their reported interacting non-immunological host factors.

### 4.1. Nonstructural Protein 1 (nsP1)

The nsP1 (535 amino acids, ~60 kDa) protein is responsible for the capping of both the positive-sense genomic viral RNA and the 26S subgenomic RNA [58,144]. Interestingly, it caps the viral RNA in a non-canonical manner where it first attaches a methyl group (hijacked from the host S-adenosyl-methionine (AdoMet)) to a GTP before transferring the methylated guanylate residue to the nsP2-processed 5′ end of the viral RNA [145,146]. For the capping to be successful, the triphosphates on the 5′ end of the viral RNA need to be cleaved by the nsP2 triphosphatase, exposing the diphosphates to allow the transfer to be complete [145]. Capping of the CHIKV RNA is believed to be a strategy to confer protection against degradation by the host exonucleases and also enable efficient translation of the viral mRNA.

To date, there is still no high resolution structural information on the nsP1 protein [147]. However, it has been suggested that the capping domain spans across at least the first 400 aa residues from the N terminus [148]. With reference to the secondary structure of the nsP1 of Sindbis virus (SINV), a related alphavirus, the CHIKV nsP1, has been speculated to carry guanylyltransferase activities [149]. However, it is important to note that the sequence (or structural) homology between SINV and CHIKV nsP1 is low, and a crystal structure and further confirmatory biochemical assays would be needed for confirmation of the guanylyltransferase activity of CHIKV nsP1. Another important function of the nsP1 protein is its monotopic interaction with the cytoplasmic side of the plasma membrane bilayer, mediated by its amphipathic alpha helix (approximately between 244 and 263 aa), discovered in a relatively well-studied, close alphavirus relative, the Semliki Forest virus [150,151]. Additionally, covalent palmitoylation of the nsP1 (417–419 aa) was discovered to be able to strengthen the association with the plasma membrane [149]. This crucial interaction allows the nsP1 to direct and anchor the replication complex to the cell plasma membrane [152]. The interactions between the nsPs have not been well explored. However, there are studies that have shown that nsP1 strongly interacts with nsP4 [153,154,155]. Similarly, there is also a lack of studies on the interaction with host proteins. The BST-2 protein was identified to be an anti-viral factor that was downregulated by the nsP1 protein to allow release of the viral particles tethered to the cell surface [142,143,156].

### 4.2. Nonstructural Protein 2 (nsP2)

The nsP2 protein is one of the more well-studied and also the largest nsP, consisting of 798 amino acids with an approximate molecular weight of 90 kDa [58,144]. So far, there are no reports of high resolution crystal structures of the entire CHIKV nsP2. However, crystals structures of the CHIKV nsP2 C terminus (~471–791 aa) (PDB code 3TRK (2011) and 4ZTB (2016)) and that of alphavirus relatives, SINV, and Venezuelan equine encephalitis virus (VEEV) are available for comparison [157,158,159]. The domains in the N terminal region, however, have been proposed via molecular modelling [160]. Starting from the N terminus, the nsP2 is hypothesized to have three structural domains [160]. The first domain (~1–167 aa) has little homology with other alphaviruses and remains unknown [160]. The subsequent two domains (~168–470 aa) possess characteristic RecA-like domains of superfamily 1 (SF1) group of helicases [160]. The remaining portion (~471–791 aa) holds the protease domain, which can be separated into two smaller sections. The first section bears a papain-like cysteine protease domain (~471–605 aa), while the last section was appears to be a non-functional Ftsj (rrmj) methyltransferase-like domain (~606–791 aa) [160].

This multi-functional nsP2 is capable of performing at least four enzymatic functions. The helicase activity is only functional when the full length nsP2 is available, even though the effector domains are found on the N terminus [160]. Truncated recombinant proteins were found to be inactive [161]. The CHIKV nsP2 is only able to unwind double-stranded RNA that have a 5′ overhang of at least 12 bp in and also only in the 5′–3′ directionality [160]. Since it possesses RecA-like domains, it is not surprising that it facilitates the complementary base-pairing of single-stranded RNA [160]. The CHIKV nsP2 has also been reported to display nucleotide triphosphatase (NTPase) activities and is able to hydrolyse all dNTPs and NTPs without any preference. However, this is observed only in full length nsP2 [160,161]. Furthermore, the activation of the NTPase of the nsP2 by RNA and DNA oligonucleotides is, again, only possible with the full length nsP2 [160]. Interestingly, the same active site used for the NTPase activity has also been found to perform RNA 5′triphosphatase activities, the third function, which mediates the cleavage of γ-phosphates from RNA substrates [161]. The last known enzymatic function is the protease activity of the nsP2, which is responsible for processing of the nsP polyprotein [162,163].

Apart from these enzymatic functions, the nsP2 has been long known to enter the host nucleus during infection, albeit with the absence of any putative nuclear localization signals [147,164]. Although the exact mechanism is still unknown, the localization of nsP2 is essential in inhibiting the host antiviral responses [165,166,167]. One interesting observation reported by Fros and colleagues is that CHIKV nsP2 can be detected in the nuclear region at 4 h post infection (h.p.i) before translocating to the cytoplasm at a later time point of 12 h.p.i [168]. One way that the CHIKV nsP2 is able to induce the shutdown of host transcription is by mediating the degradation of a catalytic subunit of the RNA polymerase II, Rpb1 [169]. The nsP2 protein has also been found to be responsible for the suppression of the host cellular translation processes without affecting viral protein translation [53]. Interactions of the alphavirus nsP2 protein with a large number of ribosomal proteins and proteins that are involved in translations have also been revealed via immunoprecipitation assays [170]. For instance, in VEEV, the ribosomal protein S6 (RpS6) was found to immunoprecipitated with nsP2 in both mammalian and insect cells [171]. Low levels of RpS6 proteins have been correlated strongly with diminished cellular translation activities [171]. However, the exact mechanism of this dephosphorylation phenomenon is still unknown. Recently, nsP2 and nsP3 were also discovered to exhibit RNA interference activity [172].

The promiscuous alphavirus nsP2 is able to bind to not only the other nsPs but also a number of host factors. The interactions with nsPs were confirmed for CHIKV through immunoprecipitation of GST and streptavidin-tagged nsPs and their various domains, and validated via a yeast two-hybird screen and ELISA [173,174]. In addition, using computational techniques, Rana and colleagues were able to propose a spatial model of the late CHIKV replication complex [173]. Bourai and colleagues performed an extensive study on the host factors interacting with the nsP2 using a yeast two-hybird screen [175]. While they identified 22 unique hits, only heterogeneous nuclear ribonucleoprotein K (hnRNP-K) and ubiquilin 4 (UBQLN4) were observed to play a significant role in CHIKV replication upon gene silencing [175]. Another host factor, the NDP52 human autophagy receptor, was shown to interact with CHIKV nsP2 and acts as a pro-viral factor in human cells [176,177]. Although the precise mechanism is still unknown, it has been postulated that the human NDP52 interacts with nsP2 in the cytoplasm to prevent the latter from localizing into the nucleus. This could in turn delay cell death, which would allow more time for the CHIKV to replicate [147]. The nsP2 is also implicated in the suppression of the unfolded protein response (UPR) triggered by the production of CHIKV envelope proteins in the endoplasmic reticulum (ER) [168]. Although the exact mechanism of action remains to be clarified, incomplete splicing of the X-box binding protein 1 (XBP1) mRNA (complete splicing is required for UPR) has been observed in cells transfected with nsP2 [168].

Being such a multifunctional protein that is involved in a number of important processes such as viral genome replication and host evasion, CHIKV nsP2 presents an intriguing and promising target for drug development. This has being aptly reviewed by Bakar and Ng [178].

### 4.3. Nonstructural Protein 3 (nsP3)

The nsP3 (530 amino acids, ~60 kDa) protein possesses three domains, a highly conserved N-terminal macro domain (~160 aa), followed by a zinc^2+^ binding domain (~165 aa) and ends with a variable “tail” region (~205 aa) [58,144,147]. The macro domain contains a ubiquitous protein module found in all living organisms including positive sense RNA viruses like alphaviruses, coronaviruses, hepatitis E virus and rubella virus [179]. The macro domain is believed to function mainly as an ADP hydrolase that removes mono or poly ADP-ribose marks on proteins. ADP-ribose marks usually occur on Asp, Glu, and Lys residues and are indicative of post-translational modifications by the poly (ADP-ribose) polymerases (PARPs) [180,181]. In experiments involving mass spectrometry, McPherson and colleagues discovered that the CHIKV nsP3 is able to recognize and hydrolyse the ADP-ribose groups from mono ADP-ribose marks that found on only Asp and Glu residues [182]. The authors went on to show that the loss of the hydrolase significantly compromised the ability of CHIKV to replicate in both baby hamster kidney and *Aedes albopictus* cells [182]. Moreover, CHIKV infectious clones encoding hydrolases with impaired activities were found to replicate more slowly in mouse neuronal NSC-34 cells with a significant decrease in fitness in neonate mouse model [182]. These results indicate that the macro domain of nsP3 proteins plays an important role in the viral replication and virulence of the CHIKV. In addition, it also suggests that the levels of ADP-ribosylation could play a major role in the anti-viral response by the host.

The zinc binding domain of CHIKV nsp3 was inferred from the crystal structure of the SINV by Shin and colleagues [158]. Mutations in this region resulted in the impairment of both the negative-sense and 26S subgenomic RNA synthesis and polyprotein processing in SINV [183,184]. Similarly, mutations in the zinc-binding region of SFV nsP3 resulted in defects in neurovirulence [185]. However, little is known about their precise mechanisms.

The stretch of conserved residues (proline–rich) on the variable “tail” region was found to be able to bind to the host amphiphysin1 and 2 proteins through the Src-homology 3 (SH3) domains found on the amphiphysin proteins [186,187]. This phenomenon is observed readily upon the transfection of the CHIKV nsP3. Although no validation was performed using CHIKV infected cells, both SFV and SINV infected cells confirmed this observation [186]. Amphiphysins are postulated to be involved in the formation of the spherules (which house replication complexes) given their ability to induce membrane curvature [147,186,188].

The nsP3 also binds to GTPase-activating protein (SH3 domain)-binding protein 1 (G3BP1) and its homolog (G3BP2). The G3BPs are found in stress granules, which are believed to have anti-viral properties. For instance, stress granules are involved in the inhibition of RNA translation [147]. By sequestering the G3BPs, the nsP3 is able to prevent stress granules from forming during infection [189,190,191]. However, the depletion of the G3BPs was found to be unfavorable for CHIKV replication [192,193,194]. The proposed pro-viral activity of the G3BPs includes aiding the switch from the translation of non-structural proteins to viral RNA replication [192,193,194]. In mosquito cells, the G3BP homolog, Rasputin (Rin), was found to also exhibit similar phenomenon [195]. However, in cell culture, the silencing of Rin did not adversely affect CHIKV replication [195]. Instead a significant reduction in total infectious viral titer was observed in *Aedes albopictus* in vivo [195]. Interestingly, Remenyi and colleagues also showed that nsP3 is closely associated with the host cellular lipid droplets and also with nsP1 [196].

Sphingosine kinase 2 (SK2) was reported to co-localize with nsP3 in nsP3 overexpression studies. In addition, SK2 also colocalizes with the CHIKV RNA [197]. Moreover, Reid and colleagues showed that upon SK2 knockdown, there was a significant reduction in infectious viral titer, suggesting a pro-viral role [197]. However, the exact mechanism still remains to be solved. Additionally, there are other host factors (Y-Box-Binding Protein 1 (YBX1), PI3K-Akt-mTOR pathway, DDX1/DDX3, and IKKβ) that were reported to be able to bind or interact with the alphavirus (SINV, SFV, and VEEV) nsP3 reviewed Lark and colleagues [198]. However, these have not been validated for CHIKV. Another pro-viral host protein found to co-immunoprecipitate with the nsP3 is the heat shock protein 90β (Hsp90β), but its exact function is unknown [199].

### 4.4. Nonstructural Protein 4 (nsP4)

The bulk of the nsP4 (611 aa, ~70 kDa) protein consists of an RNA-dependent RNA polymerase (RdRp) (~500 aa), with the characteristic GDD motif, responsible for viral RNA synthesis at its C-terminus [58,144,147]. The remaining ~100 aa at its N-terminus, despite being a relatively unknown and seemingly intrinsically disordered stretch of sequences, is still important for the normal function of the nsP4 in SINV [200]. To date, high resolution structures of the CHIKV RdRp are still not available. Recently, Chen and colleagues were able to generate a truncated but soluble, well-folded, functional RdRp catalytic subunit from *E. coli* [201]. They showed that the CHIKV RdRp has a preference for single-stranded RNA with a 5′-overhang that is of at least 4 nucleotides long [201]. In addition, the RdRp was also found to be rather sensitive to a number of detergents in comparison to the relatively resilient Dengue virus RdRp [201]. The RdRp was also able to exhibit primed extension of templates and terminal adenylyltransferase (TATase) activity regardless of the presence of any template [201].

Similar to the nsP3 protein, the nsP4 protein was found to interact with another Hsp90 protein, Hsp90α. Inhibition of Hsp90α resulted in a decrease in both viral RNA and protein levels [199]. However, the mechanism of the interaction remains to be discovered. In another study, Rathore and colleagues showed that overexpression of the CHIKV nsP4 was able to suppress the phosphorylation of eukaryotic translation initiation factor, alpha subunit (eIF2α), which in turn antagonizes the host unfolded protein response during infection [202]. On the other hand, during SINV infection, rapid phosphorylation of eIF2α was observed instead. However, the mechanism behind this interaction remains unexplored.

An in-depth review on the nsP4 functions and interactions was also recently published by Pietila and colleagues [203].

## 5. Other Host Factors That Are Involved in CHIKV Replication Cycle

In concert with the above efforts, a number of host factors have been identified via different screening methods. For instance, Paingankar and colleagues discovered that CHIKV interacts with housekeeping proteins like that actin, heat shock protein 70 (HSP70) and STAT2 (Vero-E6 cells only) via virus overlay protein binding assay (VOPBA) complemented with matrix-assisted laser desorption/ionization time of flight analysis (MALDI TOF/TOF) [204]. They went on to show that the silencing of HSP70 resulted in a significant decrease in total infectious viral titer. However, the exact mechanism remains to be solved.

Treffers and colleagues employed the use of stable isotope labeling with amino acids in cell culture (SILAC) coupled with liquid chromatography–mass spectrometry (LC-MS) to uncover the temporal dynamics of the cellular response during CHIKV infection [205]. They were able to pick up 13, 38, and 106 proteins that were differentially expressed at 8, 10, and 12 h.p.i, respectively. Moreover, majority of the proteins detected were the subunits of RNA polymerase II, and they were found to be progressively degraded [205]. This is in line with the observation that cellular transcriptional shut off occurs during CHIKV. The authors also reported four anti-viral factors (Rho family GTPase 3 (Rnd3), DEAD box helicase 56 (DDX56), polo-like kinase 1 (Plk1), and ubiquitin-conjugating enzyme E2C (UbcH10)), which were down regulated during CHIKV infection [205]. However, the exact mechanisms remain to be discovered.

A very extensive human whole genome siRNA mediated loss-of-function screen was recently performed in a bid to identify effective therapeutics against CHIKV [206]. Karlas and colleagues were able to capture 156 pro-viral and 41 antiviral host factors that affect CHIKV replication [206]. They performed pathway analysis of the identified pro-viral factors and subsequently identified 21 FDA-approved small-molecule inhibitors that were effective against CHIKV by cross referencing with specialized databases [206]. These 21 antiviral compounds were found to act on four host factors (vacuolar-type H+ ATPase (vATPase), CDC-like kinase 1 (CLK1), fms-related tyrosine kinase 4 (FLT4 or VEGFR3), and the K (lysine) acetyltransferase 5 (KAT5 or TIP60)) and two pathways (calmodulin signalling and fatty acid synthesis) [206]. Through in vivo and in vitro work, three of the inhibitors (Tivozanib, Pimozide, and 5-tetradecyloxy-2-furoic acid (TOFA)) were reported to exhibit prophylactic antiviral effects in mice [206]. In addition, when the authors combined two inhibitors (Pimozide and TOFA), each targeting the calmodoulin signalling and fatty acid synthesis pathways, respectively. The synergistic effect resulted in a therapeutic antiviral effect in both in vivo and in vitro studies [206]. However, the exact mechanism and role played by these host pathways in CHIKV replication warrants further work. Nonetheless, the work by Karlas and team showed the importance and relevance of understanding the interplay of host factors during viral infection, as well as the significant translational value that can be gained from performing basic research on the importance of host factors during CHIKV infection.

## 6. Conclusions and Perspectives

Even though a large number of host factors have been identified through these studies (Table 1), the mechanistic details of the interplay of the host factors during CHIKV infection are still lacking. The advantages of targeting host factors are plenty, as opposed to targeting just viral proteins. For instance, targeting host factors may allow inhibition of a broad-spectrum of viruses that share same host factors (Table 1). Moreover, those drugs could also be tested for synergistic effects with specific viral protein inhibitors for development of a more comprehensive treatment plan that targets multiple pathways. This therapeutic approach would also prevent the development of antiviral resistance. For instance, small molecule inhibitors that mimic the interaction sites found on GAGs, PHB, TIM, and TAMs could be developed. These would bind to CHIKV, drastically reduce the opportunity for the CHIKV to interact with the attachment factors present on the host cells and hence dampen its infectivity. Similarly, this approach could also be extended to host factors that are involved in the CHIKV replication cycle like the NDP52 host protein and given in a cocktail to patients.

A promising cocktail candidate for clinical trials would be the combination of Pimozide and TOFA concocted by Karlas and team. In drug research, though many drugs screened may have shown to possess great efficacy in vitro, many of them failed during the in vivo validation processes. On the other hand, the cocktail of Pimozide and TOFA was able exhibit impressive efficacy in both in vitro and in vivo studies. Therefore, we feel that there is great potential in this combination host-targeting drug therapy.

It should still be noted that targeting host factors comes with the risk of toxicity, especially when these host factors perform vital functions in the host cells. Design of therapeutics therefore needs to be optimized to target interactions between host and viral factors, while minimizing disruption of essential cellular processes. With the significant bottlenecks in our knowledge of basic CHIKV virology and its interacting host partners, more research is needed to understand the molecular mechanisms of the interactions between the CHIKV and its host factors. This would not only help to increase the knowledge pool but also provide more opportunities and avenues to develop, optimize, and/or speed up the production or repurposing of potential therapeutics to combat this medically important re-emerging arbovirus.

## Figures and Tables

**Table 1:** Summary of host factors known to interact with CHIKV proteins.

Viral Protein	Validated Interacting Host Factors/Pathways	Known Functions of Host Proteins	Putative Function/Interaction with CHIKV Postulated by the Authors	Examples of Interaction with Other Medically Important RNA Viruses	References
**Capsid**	**Karα4 (KPNA4)**	**Karα4 (KPNA4):** A group of proteins that transport molecules between the cytoplasm and nucleus. Able to act as either importins or exportins.	**Karα4 (KPNA4):** Binds to NLS of CHIKV capsid protein for nuclear translocation.	**Karα4 (KPNA4):** Proposed to Interact with Middle East Respiratory Syndrome (MERS) virus protein OF4b to prevent NF-kappa-B complex from entering the nucleus.	[113,207]
	**CRM1 (XPO1)**	**CRM1 (XPO1):** Major mammalian export protein that facilitates export of RNA and proteins from the nucleus to the cytoplasm.	**CRM1 (XPO1):** Pro-viral factor. Binds to the NES/of capsid, allowing exit from the nucleus.	**CRM1 (XPO1):** Proposed to bind and export the following RNA-containing viral proteins from the nucleus to the cytoplasm: human immunodeficiency virus (HIV) Rev protein cargo complex, human T-cell leukemia virus type 1 (HTLV-1) rex protein, and influenza A ribonucleoprotein complexes.	[113,208,209,210,211,212,213]
**E3**	**Furin**	**Furin:** Calcium-dependent serine endoprotease. Preferentially cleaves at sites with paired basic amino acids.	**Furin:** Cleaves the E3 protein away from the precursor E2 polyprotein.	**Furin:** Shown to be essential for H5N1, H7N1 avian influenza viruses, and Canine distemper virus (CDV). Actual mechanism unknown.	[106,118,214]
**E2**	**PHB**	**PHB:** Many reported functions, including modulation of transcription and chaperone functions in the mitochondria.	**PHB:** Possible attachment/entry factor.	**PHB:** Shown to interact with HIV-1 glycoprotein, and the binding is important for its replicative spreading in cells. Interacts with dengue virus E protein and is the first characterized receptor protein for dengue virus in insect cells. Proposed to be entry factors for hepatitis C virus.	[124,215,216,217]
	**PTPN2**	**PTPN2:** A tyrosine phosphatase that dephosphorylates protein tyrosine kinases in both nuclear and cytoplasm compartments. Involved in numerous signaling events (e.g., endocytic recycling).	**PTPN2:** Postulated to be involved in transportation of viral structural proteins to host plasma membrane.	**PTPN2:** Hepatitis C virus nonstructural 3/4A protease cleaves PTPN2 that induces a shift from host Th1 to Th2 immune response.	[128,218]
	**COL1A2**	**COL1A2:** A group 1 collagen found in most connective tissues.	**COL1A2:** Mechanism unknown.	**COL1A2:** Shown to increase infectious viral titer of Sindbis virus (SINV) and also proposed to aid in its transmission.	[128,219]
	**ACTG1**	**ACTG1:** Part of cellular trafficking machinery.	**ACTG1:** Postulated to be involved in transportation of viral structural proteins in host cell.	**ACTG1:** The human immunodeficiency virus type 1 (HIV-1) protease was found to cleave actin (ACTG1).	[128,220]
	**GAGs**	**GAGs:** A group of complex linear polysaccharides expressed on cell surface, in intracellular compartments, and also in the extracellular environment, where they are able regulate many cellular processes including (examples cell signaling, etc.).	**GAGs:** Possible attachment/entry factor.	**GAGs:** Allows binding and infection of hepatitis B virus. Attachment factor for respiratory syncytial virus (RSV), coronavirus NL63 (CoV-NL63), and the severe acute respiratory syndrome coronavirus (SARS-CoV).	[132,221,222,223,224,225]
	**hTIM1**	**hTIM1:** Involved in regulation of both innate and adaptive immune responses, engulfment of apoptotic cells, and T cell—proliferation.	**hTIM1:** Possible attachment/entry factor.	**hTIM1:** Implicated as receptors for non-enveloped hepatitis A virus and enveloped viruses such as Zaire Ebolavirus and Lake Victoria Marburgvirus.	[129,226,227,228]
	**AXL receptor tyrosine kinase**	**AXL:** Regulate and involved in many important physiological processes like cell proliferation, survival, differentiation, and migration.	**AXL:** Possible attachment/entry factor.	**AXL:** Implicated as receptors for Ebolavirus, Marburgvirus, pseudo-typed lentiviral, vaccinia virus, and Lassa virus.	[129,229,230,231]
**6K/TF**	**-**	**-**	**-**	**-**	**-**
**E1**	**COMMD1**	**COMMD1:** A proposed scaffold protein that is involved in diverse physiological processes. Able to regulate the ubiquitination and degradation of specific cellular proteins including NF-κB subunit p65.	**COMMD1:** Postulated to be involved in transport of viral structural proteins in host cell and/or involved in regulating host immune response.	**COMMD1:** Enhances latent infection of HIV-1 by stabilizing IκB-α, the inhibitor of NF-κB, and attenuating innate immune response.	[128,232,233]
	**THBS1**	**THBS1:** Adhesive glycoprotein that binds heparin. Plays a role in dentinogenesis via its anti-angiogenic properties. Also suggested to play a role in ER stress response.	**THBS1:** Mechanism unknown.	**THBS1:** Induced by hepatitis C virus (HCV) to promote the proteolytic activation TGF-β1, which promotes HCV RNA replication.	[128,234]
	**DYNC1H1**	**DYNC1H1:** Subunit of dynein complex. Integral part of cellular transport machinery across cells including neuronal cells. Plays a role in mitotic spindle and metaphase plate assembly.	**DYNC1H1:** Postulated to be involved in transport of viral structural proteins in host cell and implicated in neurological manifestations of CHIKV.	**DYNC1H1:** Aids in uncoating of HIV-1 nucleocapsids during infection. Proposed to be involved in the transport of influenza virus X-31, human foamy virus (HFV), HIV1 reverse transcription complexes (RTC), herpes simplex virus type 1, and Mokola virus.	[128,235,236]
	**ATP1B3**	**ATP1B3:** Part of the sodium/potassium-transporting ATPase that maintains electrochemical gradient and is important for osmoregulation.	**ATP1B3:** Probably facilitates fusion of viral envelope to host membrane during viral entry.	**ATP1B3:** Shown to inhibit enterovirus 71 (EV71) replication by up-regulating type-I interferon production. Proposed to be a pro-viral factor for HIV-1 by accelerating the degradation of BST-2.	[128,235,236]
	**BST-2**	**BST-2:** Lipid-raft associated protein that is part of the antiviral response pathway. Blocks the release of many enveloped mammalian virus by tethering the mature virions to the cell plasma membrane of the infected cells.	**BST-2:** Proposed to restrict virus release by latching onto the CHIKV E1 protein.	**BST-2:** Restricts Lassa virus replication and release. Restricts viral like particle (VLP) release of the following viruses: vesicular stomatitis virus (VSV), hepatitis C virus (HCV), Kaposi’s sarcoma-associated herpesvirus (KSHV), human immunodeficiency virus 1 (HIV-1), ebola virus, Machupo virus MACV) Nipah virus, Zaire ebolavirus (ZEBOV), Lake Victoria marburgvirus (MARV), Rift Valley fever virus (RVFV), cowpox virus (CPXV), and influenza virus.	[142,143,237,238,239,240,241,242,243,244]
**nsP1**	**BST-2**	**BST-2:** See above.	**BST-2:** nsP1 reverses BST-2 ability to restrict virus release by down-regulating the latter’s expression.	**BST-2:** See above.	[142,143,156]
**nsP2**	**Rpb1**	**Rpb1:** A catalytic subunit of the RNA polymerase II complex that catalyses RNA transcription.	**Rpb1:** Does not get degraded by the CHIKV nsP2 proteins. Instead is degraded via nsP2 mediated ubiquitination.	**Rpb1:** Same observations were found in Sindbis, Semliki Forest virus (old world alphaviruses).	[169]
	**SFRS3**/SRp20 (Serine and Arginine Rich Splicing Factor 3)	**SFRS3 (SRp20):** RNA splicing factor, aids in exon-inclusion during alternative splicing. Involved in mRNA nuclear export.	**SFRS3 (SRp20):** Mechanism unknown.	**SFRS3 (SRp20):** Proposed to be crucial for IRES-mediated translation in poliovirus.	[175,245]
	**VIM** (Vimentin), **TACC3** (transforming, acidic coiled-coil containing protein 3), **CEP55** (centrosomal protein 55 kDa), and **KLC4** (kinesin light chain 4)	**VIM, TACC3, CEP55, and KLC4:** Cytoskeletal components.	**VIM, TACC3, CEP55 and KLC4:** Proposed to be hijacked by nsP2 for transport into the infected cells. Interaction with CHIKV nsP3 was also reported and is proposed to aid in the anchorage of the replication complex.	**VIM:** Proposed to be involved in the distribution and acidification of endosomes, allowing successful release of influenza A viral genome. **TACC3, CEP55, KLC4:** Not reported.	[175,246,247]
	**ASCC2** (activating signal cointegrator 1 complex subunit 2), **TRIM27** (tripartite motif 27), **MRFAP1L1/MRG15** (Morf4 family-associated protein 1-like 1), **EWSR1** (Ewing sarcoma breakpoint region 1), **IKZF1** (IKAROS family zinc finger 1) and **ZBTB43** (zinc finger and BTB domain-containing 43)	**ASCC2:** Proposed to regulate/involved in DNA transcription and repair. **TRIM27:** Represses gene transcription through ubiquitination. **MRFAP1L1/MRG15:** Proposed to regulate transcription by interacting with both the retinoblastoma tumor suppressor (Rb) and a nuclear protein PAM14 (protein associated with MRG, 14 kDa). **EWSR1:** Proposed to be involved in gene expression, cell signaling, and RNA processing and transport. **IKZF1:** A transcription factor that is associated with chromatin remodeling. **ZBTB43:** Proposed to suppress transcription of Blimp1 [PR domain zinc finger protein 1] (a transcription factor for various innate and adaptive immune cell types).	**ASCC2, TRIM27, MRF4P1L1, EWSR1, IKZF1, and ZBTB43:** Mechanism unknown.	**ASCC2:** Not reported. **TRIM27:** Interacts and is degraded by the immediate early protein ICP0 of the herpes simplex virus 1 (HSV-1). However, depletion of TRIM27 in cells results in reduced virus titer. **MRFAP1L1:** Not reported. **EWSR1:** Binds directly with the *cis*-acting replication element (CRE) of hepatitis C virus. Depletion of EWSR1 impairs HCV viral replication and reduced virus titers without affecting translation. **IKZF1:** Not reported. **ZBTB43:** Not reported.	[175,248,249,250]
	**HNRNPK**	**HNRNPK:** Binds to cytidine-rich pre-mRNA. Proposed to play a role in hnRNA metabolism. Plays an important role response to DNA damage via P53 pathway. Able to activate and repress transcription.	**HNRNPK:** Mechanism unknown.	**HNRNPK:** Proposed restrict HCV replication by limiting the availability of the HCV RNA for packaging into virions. Demonstrated to support vesicular stomatitis virus (VSV) infection via (1) suppression of apoptosis in infected cells, (2) inhibiting antiviral protein expression, and (3) supporting the expression of several cellular proteins necessary for the virus.	[175,251,252]
	**TTC7B** (Tetratricopeptide repeat domain 7B)	**TTC7B:** Part of a complex that regulates and localizes phosphatidylinositol 4-kinase. (PI4K) to the cell plasma membrane.	**TTC7B:** Aids nsP2 in shutting off host cellular processes.	**TTC7B:** Dengue 2 virus induce expressions of proteins that contain tetratricopeptide repeats (TTC).	[175,253,254]
	**UBQLN4, RCHY1** (ring finger and CHY zinc finger domain-containing 1), and **WWP1** (WW domain-containing E3 ubiquitin protein ligase 1)	**UBQLN4, RCHY1, and WWP1:** Involved in autophagy and/or protein degradation	**RCHY1 and WWP1:** Mechanism unknown. **UBQLN4:** Pro-viral factor. Mechanism unknown.	**RCHY1:** Interacts with SARS nonstructural protein 3 to increase degradation of P53, which is involved in innate antiviral immunity. **WWP1:** Aids in budding of ebola virus VP40 matrix protein via ubiquitination of the matrix proteins. **UBQLN4:** Shown to interact with small hydrophobic (SH) protein of mumps virus and relocate them to 20S proteasome, possibly for proteosomal degradation.	[175,255,256,257]
	**PDK2** (pyruvate dehydrogenase kinase, isozyme 2), **RBM12B** (RNA-binding motif protein 12B), **GFAP** (glial fibrillary acidic protein), and **TPR** (translocated promoter region [to activated MET oncogene])	**PDK2:** Phosphorylate pyruvate dehydrogenase subunits to regulate glucose and fatty acid metabolism and homeostasis. Regulate cell proliferation and delay apoptosis when cells are under oxidative stress. **RBM12B:** RNA-binding protein **GFAP:** A type of intermediate filament (class-III) and is a cell-specific marker that helps to differentiate astrocytes from other glial cells during the development of the central nervous system. **TPR:** A scaffolding component found on the nuclear face of the nuclear pore complex. Allows transport of proteins and mRNAs out of the nucleus. Also aids in perinuclear chromatin distribution.	**PDK2, RBM12B, GFAP, and TPR:** Mechanism unknown.	**PDK2:** Not reported. **RBM12B:** Not reported. **GFAP:** Measles virus disrupts the glial-fibrillary-acidic protein filament (GFAP) network in Astrocytoma Cell Line (U-251). Exact function is unknown. **TPR:** Suppressed by the Avian reovirus (ARV) protein p17 Knock-down of TPR has shown to increase ARV titer.	[175,258,259,260]
	**NDP52/CALCOCO2** (calcium-binding and coiled-coil domain-containing protein 2)	**NDP52:** Involved in autophagy; recruits and degrades intracellular pathogens and is able to inhibit proliferations of pathogens like Salmonella.	**NDP52:** Seems to be able to recruit CHIKV nsP2 and LC3C to the trans-Golgi network that contains double-stranded RNA and other nsPs. This is postulated to allow formation of the replication complexes, thereby promoting viral infection.	**NDP52:** Proposed to modulate innate immune response upon interacting with influenza virus protein PB1-F2.	[175,176,177,261,262]
**nsP3**	**PI3K-Akt-mTOR pathway**	**PI3K-Akt-mTOR pathway:** Regulates cell cycle and is directly involved in cellular proliferation, quiescence, and cancer.	**PI3K-Akt-mTOR pathway:** Drives the internalization of the replication complex.	**PI3K-Akt-mTOR pathway:** Essential for survival of host and virus (Hepatitis C Virus, Vaccinia, and Cowpox Virus).	[75,263,264]
	**G3BP1 & G3BP2**	**G3BP1:** Marker for stress granules and may be an effector for stress granule assembly. Able to unwind DNA and RNA. **G3BP2:** Postulated to be a scaffold protein which could transport mRNA.	**G3BP1 & G3BP2:** Colocalizes with nsP2 and nsP3. Depletion of both proteins results in reduction of viral RNA (especially the negative sense RNA), proteins, and infectious titer. Authors proposed that the G3BPs could mediate the switch from translation to amplification of viral genome.	**G3BP1:** Knock-down of G3BP1 results an increase in HIV-1 viral titer only in primary T cells and macrophages. Found to interact with HIV-1 RNA in the cytoplasm. G3BP1 is proposed to sequester viral RNA transcripts, preventing translation and packaging. **G3BP1 and G3BP2:** DENV-2 non-coding subgenomic flaviviral RNA (sfRNA) was found to bind to both G3BP1 and G3BP2 and inhibit their antiviral activities.	[192,265,266]
	**SK2**	**SK2:** A lipid kinase that phosphorylates sphingosine to form sphingosine 1-phosphate (SPP). Involved in cell differentiation, growth, and host immunity.	**SK2:** Colocalizes with CHIKV RNA and nsPs. Knock-down of SK2 inhibits CHIKV infection.	**SK2:** Not reported.	[197]
	**Hsp90β**	**Hsp90β:** A cytoplasmic isoform of HSP-90s molecular chaperons that is constitutively expressed. They are able to modulate different cellular processes (e.g., refold proteins) to maintain cellular homeostasis.	**Hsp90β:** Mechanism unclear. Proposed to have an ancillary role in CHIKV replication. On a side note, Hsp90 is proposed to stabilize CHIKV nsP2 during infection.	**Hsp90β:** Proposed to be the binding receptor for Japanese encephalitis virus (JEV). Proposed to facilitate assembly of enterovirus 71 viral particles. Hepatitis B virus polymerase interacts with Hsp90β to suppress NF-kB signaling.	[199,267,268,269,270]
**nsP4**	**LCP1** (Lymphocyte cytosolic protein 1/l-Plastin)	**LCP1:** Binds to actin and aids in activation of T-cells during co-stimulation through other receptors.	**LCP1:** Mechanism unknown.	**LCP1:** Not reported.	[175]
	**Hsp90α**	**Hsp90α:** A cytoplasmic isoform of HSP-90s molecular chaperons that is produced during cell stress response. They are able to modulate different cellular processes (e.g., refold proteins) to maintain cellular homeostasis.	**Hsp90α:** Proposed to help in stabilizing CHIKV nsP4 and aid in formation of CHIKV replication complex in the cytosol. Knock-down of Hsp90α resulted in a decrease in viral RNA.	**Hsp90α:** Not reported.	[199]
	**eIF2α**	**eIF2α:** A eukaryotic initiation factor that is essential for initiating translation.	**eIF2α:** nsP4 suppresses the serine-51 phosphorylation of eIF2α, which in turn regulates the PERK pathway, allowing the CHIKV to overcome the host unfolded protein response machinery.	**eIF2α:** The presence of the three rotavirus proteins, VP2, NSP2, and NSP5, induces the phosphorylation of eIF2α. However, formation of stress granules (which stalls translation) was inhibited. HSV utilizes its neurovirulence factor ICP34.5 to dephosphorylate eIF2α.	[202,271,272]

## References

[1] Silva L.A., Dermody T.S. (2017). Chikungunya virus: Epidemiology, replication, disease mechanisms, and prospective intervention strategies. J. Clin. Investig..

[2] Waggoner J.J., Pinsky B.A. (2015). How Great is the Threat of Chikungunya Virus?. Expert Rev. Anti Infect. Ther..

[3] Sreekumar E., Issac A., Nair S., Hariharan R., Janki M.B., Arathy D.S., Regu R., Mathew T., Anoop M., Niyas K.P. (2010). Genetic characterization of 2006–2008 isolates of Chikungunya virus from Kerala, South India, by whole genome sequence analysis. Virus Genes.

[4] Robinson M.C. (1957). An epidemic of virus disease in Southern Province, Tanganyika Territory, in 1952–1953. Trans. R. Soc. Trop. Med. Hyg..

[5] Powers A.M., Brault A.C., Tesh R.B., Weaver S.C. (2000). Re-emergence of chikungunya and o’nyong-nyong viruses: Evidence for distinct geographical lineages and distant evolutionary relationships. J. Gen. Virol..

[6] Powers A.M., Logue C.H. (2007). Changing patterns of chikunya virus: Re-emergence of a zoonotic arbovirus. J. Gen. Virol..

[7] Schuffenecker I., Iteman I., Michault A., Murri S., Frangeul L., Vaney M.C., Lavenir R., Pardigon N., Reynes J.M., Pettinelli F. (2006). Genome microevolution of chikungunya viruses causing the Indian Ocean outbreak. PLoS Med..

[8] Volk S.M., Chen R., Tsetsarkin K.A., Adams A.P., Garcia T.I., Sall A.A., Nasar F., Schuh A.J., Holmes E.C., Higgs S. (2010). Genome-Scale Phylogenetic Analyses of Chikungunya Virus Reveal Independent Emergences of Recent Epidemics and Various Evolutionary Rates. J. Virol..

[9] Das T., Jaffar-Bandjee M.C., Hoarau J.J., Trotot P.K., Denizot M., Lee-Pat-Yuen G., Sahoo R., Guiraud P., Ramful D., Robin S. (2010). Chikungunya fever: CNS infection and pathologies of a re-emerging arbovirus. Prog. Neurobiol..

[10] Carey D.E. (1971). Chikungunya and dengue: A case of mistaken identity?. J. Hist. Med. Allied Sci..

[11] Velasco J.M., Valderama M.T., Lopez M.N., Chua D., Latog R., Roque V., Corpuz J., Klungthong C., Rodpradit P., Hussem K. (2015). Chikungunya virus infections among patients with dengue-like illness at a tertiary care hospital in the Philippines, 2012–2013. Am. J. Trop. Med. Hyg..

[12] Sudeep A.B., Parashar D. (2008). Chikungunya: An overview. J. Biosci..

[13] Staples J.E., Breiman R.F., Powers A.M. (2009). Chikungunya fever: An epidemiological review of a re-emerging infectious disease. Clin. Infect. Dis..

[14] Fritel X., Rollot O., Gérardin P., Gaüzère B.A., Bideault J., Lagarde L., Dhuime B., Orvain E., Cuillier F., Ramful D. (2010). Chikungunya virus infection during pregnancy, Réunion, France, 2006. Emerg. Infect. Dis..

[15] Vazeille M., Moutailler S., Coudrier D., Rousseaux C., Khun H., Huerre M., Thiria J., Dehecq J.S., Fontenille D., Schuffenecker I. (2007). Two Chikungunya isolates from the outbreak of La Reunion (Indian Ocean) exhibit different patterns of infection in the mosquito, Aedes albopictus. PLoS ONE.

[16] Wahid B., Ali A., Rafique S., Idrees M. (2017). Global expansion of chikungunya virus: Mapping the 64-year history. Int. J. Infect. Dis..

[17] Madariaga M., Ticona E., Resurrecion C. (2016). Chikungunya: Bending over the Americas and the rest of the world. Braz. J. Infect. Dis..

[18] Rezza G., Nicoletti L., Angelini R., Romi R., Finarelli A., Panning M., Cordioli P., Fortuna C., Boros S., Magurano F. (2007). Infection with chikungunya virus in Italy: An outbreak in a temperate region. Lancet.

[19] WHO (2018). Chikungunya. http://www.who.int/mediacentre/factsheets/fs327/en/.

[20] MOH (2016). Communicable Diseases Surveillance in Singapore 2015.

[21] Gallian P., Leparc-Goffart I., Richard P., Maire F., Flusin O., Djoudi R., Chiaroni J., Charrel R., Tiberghien P., de Lamballerie X. (2017). Epidemiology of Chikungunya Virus Outbreaks in Guadeloupe and Martinique, 2014: An Observational Study in Volunteer Blood Donors. PLoS Negl. Trop. Dis..

[22] Davidas M., Dorléans F., Septfons A., Najioullah F., Paty M.-C., Guyomard S., Cabié A., Herrmann-Storck C., Preira A., Schepers K.M. (2018). Outbreak of Chikungunya in the French Caribbean Islands of Martinique and Guadeloupe: Findings from a Hospital-Based Surveillance System (2013–2015). Am. J. Trop. Med. Hyg..

[23] European Centre for Disease Prevention and Control (2017). Clusters of Autochthonous Chikungunya Cases in Conclusions and Options for Response.

[24] Roiz D., Boussès P., Simard F., Paupy C., Fontenille D. (2015). Autochthonous Chikungunya transmission and extreme climate events in Southern France. PLoS Negl. Trop. Dis..

[25] Fourie E.D., Morrison J.G. (1979). Rheumatoid arthritic syndrome after chikungunya fever. S. Afr. Med. J..

[26] Couderc T., Chrétien F., Schilte C., Disson O., Brigitte M., Guivel-Benhassine F., Touret Y., Barau G., Cayet N., Schuffenecker I. (2008). A mouse model for Chikungunya: Young age and inefficient type-I interferon signaling are risk factors for severe disease. PLoS Pathog..

[27] Ozden S., Huerre M., Riviere J.-P., Coffey L.L., Afonso P.V., Mouly V., de Monredon J., Roger J.-C., el Amrani M., Yvin J.-L. (2007). Human Muscle Satellite Cells as Targets of Chikungunya Virus Infection. PLoS ONE.

[28] Couderc T., Lecuit M. (2009). Focus on Chikungunya pathophysiology in human and animal models. Microbes Infect..

[29] Thiberville S.D., Boisson V., Gaudart J., Simon F., Flahault A., de Lamballerie X. (2013). Chikungunya Fever: A Clinical and Virological Investigation of Outpatients on Reunion Island, South-West Indian Ocean. PLoS Negl. Trop. Dis..

[30] Dupuis-Maguiraga L., Noret M., Brun S., le Grand R., Gras G., Roques P. (2012). Chikungunya disease: Infection-associated markers from the acute to the chronic phase of arbovirus-induced arthralgia. PLoS Negl. Trop. Dis..

[31] Torres J.R., Falleiros-Arlant L.H., Dueñas L., Pleitez-Navarrete J., Salgado D.M., del Castillo J.B. (2016). Congenital and perinatal complications of chikungunya fever: A Latin American experience. Int. J. Infect. Dis..

[32] Touret Y., Randrianaivo H., Michault A., Schuffenecker I., Kauffmann E., Lenglet Y., Barau G., Fourmaintraux A. (2006). Transmission materno-fœtale précoce du virus Chikungunya. Presse Med..

[33] Lenglet A.Y., Barau G., Robillard P.-Y., Randrianaivo H., Michault A., Bouveret A., Gérardin P., Boumahni B., Touret Y., Kauffmann E. (2006). Travail original Infection à Chikungunya chez la femme enceinte et risque de transmission materno-fœtale. J. Gynécol. Obstét. Biol. Reprod..

[34] Taksande A., Vilhekar K.Y. (2015). Neonatal chikungunya infection. J. Prev. Infect. Control.

[35] Burt F.J., Chen W., Miner J.J., Lenschow D.J., Merits A., Schnettler E., Kohl A., Rudd P.A., Taylor A., Herrero L.J. (2017). Chikungunya virus: An update on the biology and pathogenesis of this emerging pathogen. Lancet Infect. Dis..

[36] Manimunda S.P., Vijayachari P., Uppoor R., Sugunan A.P., Singh S.S., Rai S.K., Sudeep A.B., Muruganandam N., Chaitanya I.K., Guruprasad D.R. (2010). Clinical progression of chikungunya fever during acute and chronic arthritic stages and the changes in joint morphology as revealed by imaging. Trans. R. Soc. Trop. Med. Hyg..

[37] Sissoko D., Malvy D., Ezzedine K., Renault P., Moscetti F., Ledrans M., Pierre V. (2009). Post-epidemic Chikungunya disease on reunion island: Course of rheumatic manifestations and associated factors over a 15-month period. PLoS Negl. Trop. Dis..

[38] Rulli N.E., Guglielmotti A., Mangano G., Rolph M.S., Apicella C., Zaid A., Suhrbier A., Mahalingam S. (2009). Amelioration of alphavirus-induced arthritis and myositis in a mouse model by treatment with bindarit, an inhibitor of monocyte chemotactic proteins. Arthritis Rheum..

[39] Labadie K. (2010). Chikungunya disease in nonhuman primates leads to long-term viral persistence in macrophages. J. Clin. Investig..

[40] Kaur P., Chu J.J.H. (2013). Chikungunya virus: An update on antiviral development and challenges. Drug Discov. Today.

[41] De Lamballerie X., Ninove L., Charrel R. (2009). Antiviral Treatment of Chikungunya Virus Infection. Infect. Disord. Drug Targets.

[42] Brighton S.W. (1984). Chloroquine phosphate treatment of chronic Chikungunya arthritis. An open pilot study. S. Afr. Med. J..

[43] Ravichandran R., Manian M. (2008). Ribavirin therapy for Chikungunya arthritis. J. Infect. Dev. Ctries..

[44] Dorr M.P.P., Westby M., Dobbs S., Griffin P., Irvine B., Macartney M., Mori J., Rickett G., Smith-Burchnell C., Napier C. (2005). Maraviroc (UK-427,857), a Potent, Orally Bioavailable, and Selective Small-Molecule Inhibitor of Chemokine Receptor CCR5 with Broad-Spectrum Anti-Human Immunodeficiency Virus Type 1 Activity. Antimicrob. Agents Chemother..

[45] De Wilde A.H., Zevenhoven-Dobbe J.C., van der Meer Y., Thiel V., Narayanan K., Makino S., Snijder E.J., van Hemert M.J. (2011). Cyclosporin A inhibits the replication of diverse coronaviruses. J. Gen. Virol..

[46] Barik S. (2012). New treatments for influenza. BMC Med..

[47] Prichard M.N., Whitley R.J. (2014). The development of new therapies for human herpesvirus 6. Curr. Opin. Virol..

[48] Price N.B., Prichard M.N. (2011). Progress in the development of new therapies for herpesvirus infections. Curr. Opin. Virol..

[49] Bekerman E., Einav S. (2015). Combatting Emerging Viral Threats. Science.

[50] Lanford R.E., Hildebrandt-eriksen E.S., Petri A., Persson R., Lindow M., Munk M.E., Kauppinen S., Ørum H. (2012). Therapeutic silencing of microRNA-122 in primates with chronic hepatitis C virus infection. Science.

[51] Gilmore J.H. (2008). Curing a viral infection by targeting the host: The example of cyclophilin inhibitors. North.

[52] Yang G., Sau C., Lai W., Cichon J., Li W. (2015). Productive Replication of Ebola Virus Is Regulated by the c-Abl1 Tyrosine Kinase. Sci. Transl. Med..

[53] Strauss J.H., Strauss E.G. (1994). The alphaviruses: Gene expression, replication, and evolution. Microbiol. Rev..

[54] Simizu B., Yamamoto K., Hashimoto K., Ogata T. (1984). Structural proteins of Chikungunya virus. J. Virol..

[55] Higashi N., Matsumoto A., Tabata K., Nagatomo Y. (1967). Electron microscope study of development of Chikungunya virus in green monkey kidney stable (VERO) cells. Virology.

[56] Solignat M., Gay B., Higgs S., Briant L., Devaux C. (2009). Replication cycle of chikungunya: A re-emerging arbovirus. Virology.

[57] Leung J.Y.S., Ng M.M.L., Chu J.J.H. (2011). Replication of alphaviruses: A review on the entry process of alphaviruses into cells. Adv. Virol..

[58] Khan A.H., Morita K., Del M., Parquet C., Hasebe F., Mathenge E.G.M., Igarashi A. (2002). Complete nucleotide sequence of chikungunya virus and evidence for an internal polyadenylation site. J. Gen. Virol..

[59] Dash P.K., Santhosh S.R., Shukla J., Dhanwani R., Rao P.V.L., Parida M.M., Liu D.Y. (2010). Chapter 29 Chikungunya Virus. Molecular Detection of Human Viral Pathogens.

[60] Voss J.E., Vaney M.C., Duquerroy S., Vonrhein C., Girard-Blanc C., Crublet E., Thompson A., Bricogne G., Rey F.A. (2010). Glycoprotein organization of Chikungunya virus particles revealed by X-ray crystallography. Nature.

[61] Schwartz O., Albert M.L. (2010). Biology and pathogenesis of chikungunya virus. Nat. Rev. Microbiol..

[62] Sourisseau M., Schilte C., Casartelli N., Trouillet C., Guivel-Benhassine F., Rudnicka D., Sol-Foulon N., le Roux K., Prevost M.C., Fsihi H. (2007). Characterization of reemerging chikungunya virus. PLoS Pathog..

[63] Bernard E., Solignat M., Gay B., Chazal N., Higgs S., Devaux C., Briant L. (2010). Endocytosis of chikungunya virus into mammalian cells: Role of clathrin and early endosomal compartments. PLoS ONE.

[64] Ooi Y.S., Stiles K.M., Liu C.Y., Taylor G.M., Kielian M. (2013). Genome-Wide RNAi Screen Identifies Novel Host Proteins Required for Alphavirus Entry. PLoS Pathog..

[65] Van Duijl-Richter M., Hoornweg T., Rodenhuis-Zybert I., Smit J. (2015). Early Events in Chikungunya Virus Infection—From Virus CellBinding to Membrane Fusion. Viruses.

[66] Hoornweg T.E., van Duijl-Richter M.K.S., Nuñez N.V.A., Albulescu I.C., van Hemert M.J., Smit J.M. (2016). Dynamics of Chikungunya Virus Cell Entry Unraveled by Single-Virus Tracking in Living Cells. J. Virol..

[67] Lee R.C.H., Hapuarachchi H.C., Chen K.C., Hussain K.M., Chen H., Low S.L., Ng L.C., Lin R., Ng M.M.L., Chu J.J.H. (2013). Mosquito Cellular Factors and Functions in Mediating the Infectious entry of Chikungunya Virus. PLoS Negl. Trop. Dis..

[68] Van Duijl-Richter M.K.S., Blijleven J.S., van Oijen A.M., Smit J.M. (2015). Chikungunya virus fusion properties elucidated by single-particle and bulk approaches. J. Gen. Virol..

[69] Singh I., Helenius A. (1992). Role of ribosomes in Semliki Forest virus nucleocapsid uncoating. J. Virol..

[70] Li G., Rice C.M. (1993). The signal for translational readthrough of a UGA codon in Sindbis virus RNA involves a single cytidine residue immediately downstream of the termination codon. J. Virol..

[71] Utt A., Quirin T., Saul S., Hellström K., Ahola T., Merits A. (2016). Versatile trans-replication systems for chikungunya virus allow functional analysis and tagging of every replicase protein. PLoS ONE.

[72] Frolova E.I., Gorchakov R., Pereboeva L., Atasheva S., Frolov I. (2010). Functional Sindbis Virus Replicative Complexes Are Formed at the Plasma Membrane. J. Virol..

[73] Kujala P., Ehsani N., Vihinen H., Ika A. (2001). Biogenesis of the Semliki Forest Virus RNA Replication Complex. Society.

[74] Spuul P., Balistreri G., Kaariainen L., Ahola T. (2010). Phosphatidylinositol 3-Kinase-, Actin-, and Microtubule-Dependent Transport of Semliki Forest Virus Replication Complexes from the Plasma Membrane to Modified Lysosomes. J. Virol..

[75] Thaa B., Biasiotto R., Eng K., Neuvonen M., Götte B., Rheinemann L., Mutso M., Utt A., Varghese F., Balistreri G. (2015). Differential Phosphatidylinositol-3-Kinase-Akt-mTOR Activation by Semliki Forest and Chikungunya Viruses Is Dependent on nsP3 and Connected to Replication Complex Internalization. J. Virol..

[76] Takkinen K., Peranen J., Kaariainen L. (1991). Proteolytic processing of semliki forest virus-specif non-structural polyprotein. J. Gen. Virol..

[77] De Groot R.J., Hardy W.R., Shirako Y., Strauss J.H. (1990). Cleavage-site preferences of Sindbis. EMBO J..

[78] Lemm J.A., Rumenapf T., Strauss E.G., Strauss J.H., Rice C.M. (1994). Polypeptide requirements for assembly of functional Sindbis virus replication complexes: A model for the temporal regulation of minus- and plus-strand RNA synthesis. EMBO J..

[79] Indra D., Sawicki S.G., Sawicki D.L. (1996). Sindbis virus RNA-negative mutants that fail to convert from minus-strand to plus-strand synthesis: Role of the nsP2 protein. J. Virol..

[80] Shirako Y., Strauss J.H. (1994). Regulation of Sindbis Virus RNA Replication: Uncleaved P123 and nsP4 Function in Minus-Strand RNA Synthesis, whereas Cleaved Products from P123 Are Required for Efficient Plus-Strand RNA Synthesis. J. Virol..

[81] Kim K.H., Rümenapf T., Strauss E.G., Strauss J.H. (2004). Regulation of Semliki Forest virus RNA replication: A model for the control of alphavirus pathogenesis in invertebrate hosts. Virology.

[82] Melancont P., Garoff H. (1987). Processing of the Semliki Forest virus structural polyprotein: Role of the capsid protease. J. Virol..

[83] Aliperti G., Schlesinger M.J. (1978). Evidence for an autoprotease activity of sindbis virus capsid protein. Virology.

[84] Garoff H., Simons K., Dobberstein B. (1978). Assembly of the Semliki Forest Virus Membrane Glycoproteins in the Membrane of the Endoplasmatic Reticulum in Vitro. J. Mol. Biol..

[85] Owen K.E., Kuhn R.J. (1996). Identification of a region in the Sindbis virus nucleocapsid protein that is involved in specificity of RNA encapsidation. J. Virol..

[86] Weiss B., Nitschko H., Ghattas I., Wright R., Schlesinger S. (1989). Evidence for specificity in the encapsidation of Sindbis virus RNAs. J. Virol..

[87] Firth A.E., Chung B.Y.W., Fleeton M.N., Atkins J.F. (2008). Discovery of frameshifting in Alphavirus 6K resolves a 20-year enigma. Virol. J..

[88] Snyder J.E., Kulcsar K.A., Schultz K.L.W., Riley C.P., Neary J.T., Marr S., Jose J., Griffin D.E., Kuhn R.J. (2013). Functional Characterization of the Alphavirus TF Protein. J. Virol..

[89] Lobigs M., Zhao H.X., Garoff H. (1990). Function of Semliki Forest virus E3 peptide in virus assembly: Replacement of E3 with an artificial signal peptide abolishes spike heterodimerization and surface expression of E1. J. Virol..

[90] Ryan C., Ivanova L., Schlesinger M.J. (1998). Effects of site-directed mutations of transmembrane cysteines in sindbis virus E1 and E2 glycoproteins on palmitylation and virus replication. Virology.

[91] Ivanova L., Schlesinger M.J. (1993). Site-directed mutations in the Sindbis virus E2 glycoprotein identify palmitoylation sites and affect virus budding. J. Virol..

[92] Ziemiecki A., Garoff H., Simons K. (1980). Formation of the Semlike Forest membrane glycoprotein complexes in the infected cell. J.Gen. Virol..

[93] De Curtis I., Simons K. (1988). Dissection of Semliki Forest virus glycoprotein delivery from the trans-Golgi network to the cell surface in permeabilized BHK cells. Proc. Natl. Acad. Sci. USA.

[94] Sariola M., Saraste J., Kuismanen E. (1995). Communication of post-Golgi elements with early endocytic pathway: Regulation of endoproteolytic cleavage of Semliki Forest virus p62 precursor. J. Cell Sci..

[95] Ekström M., Liljeström P., Garoff H. (1994). Membrane protein lateral interactions control Semliki Forest virus budding. EMBO J..

[96] Griffiths G., Fuller S.D., Back R., Hollinshead M., Pfeiffer S., Simons K. (1989). The dynamic nature of the Golgi complex. J. Cell Biol..

[97] Griffiths G., Quinn P., Warren G. (1983). Dissection of the golgi-complex.1. Monensin inhibits the transport of viral membrane-proteins from medial to trans golgi cisternae in baby hamster-kidney cells infected with Semliki Forest virus. J. Cell Biol..

[98] Soonsawad P., Xing L., Milla E., Espinoza J.M., Kawano M., Marko M., Hsieh C., Furukawa H., Kawasaki M., Weerachatyanukul W. (2010). Structural Evidence of Glycoprotein Assembly in Cellular Membrane Compartments prior to Alphavirus Budding. J. Virol..

[99] Chen K.C., Kam Y.-W., Lin R.T.P., Ng M.M.-L., Ng L.F., Chu J.J.H. (3013). Comparative analysis of the genome sequences and replication profiles of chikungunya virus isolates within the East, Central and South African (ECSA) lineage. Virol. J..

[100] Sjo M., Garoff H., Sjöberg M. (2003). Interactions between the transmembrane segments of the alphavirus E1 and E2 proteins play a role in virus budding and fusion. J. Virol..

[101] Ng M.L., Tan S.H., Chu J.J.H. (2001). Transport and budding at two distinct sites of visible nucleocapsids of West Nile (Sarafend) virus. J. Med. Virol..

[102] Sharma R., Fatma B., Saha A., Bajpai S., Sistla S., Dash P.K., Parida M., Kumar P., Tomar S. (2016). Inhibition of chikungunya virus by picolinate that targets viral capsid protein. Virology.

[103] Thomas S., Rai J., John L., Günther S., Drosten C., Pützer B.M., Schaefer S. (2010). Functional dissection of the alphavirus capsid protease: Sequence requirements for activity. Virol. J..

[104] Garmashova N., Gorchakov R., Volkova E., Paessler S., Frolova E., Frolov I. (2007). The Old World and New World Alphaviruses Use Different Virus-Specific Proteins for Induction of Transcriptional Shutoff. J. Virol..

[105] Hong E.M., Perera R., Kuhn R.J. (2006). Alphavirus Capsid Protein Helix I Controls a Checkpoint in Nucleocapsid Core Assembly. J. Virol..

[106] Metz S.W., Pijlman G.P., Okeoma C.M. (2016). Function of Chikungunya Virus Structural Proteins. Chikungunya Virus Advances in Biology, Pathogenesis, and Treatment.

[107] Weiss B., Geigenmüller-gnirke U., Schlesinger S. (1994). Interactions between sindbis virus RNAs and a 68 amino acid derivative of the viral capsid protein further defines the capsid binding site. Nucleic Acids Res..

[108] Linger B.R., Kunovska L., Kuhn R.J., Golden B.L. (2004). Sindbis virus nucleocapsid assembly: RNA folding promotes capsid protein dimerization. RNA.

[109] Sokoloski K.J., Nease L.M., May N.A., Gebhart N.N., Jones C.E., Morrison T.E., Hardy R.W. (2017). Identification of Interactions between Sindbis Virus Capsid Protein and Cytoplasmic vRNA as Novel Virulence Determinants. PLoS Pathog..

[110] Choi H.K., Tong L., Minor W., Dumas P., Boege U., Rossmann M.G., Wengler G. (1991). Structure of Sindbis virus core protein reveals a chymotrypsin-like serine proteinase and the organization of the virion. Nature.

[111] Aggarwal M., Sharma R., Kumar P., Parida M., Tomar S. (2015). Kinetic characterization of trans-proteolytic activity of Chikungunya virus capsid protease and development of a FRET-based HTS assay. Sci. Rep..

[112] Sharma R., Kesari P., Kumar P., Tomar S. (2018). Structure-function insights into chikungunya virus capsid protein: Small molecules targeting capsid hydrophobic pocket. Virology.

[113] Thomas S., Rai J., John L., Schaefer S., Pützer B.M., Herchenröder O. (2013). Chikungunya virus capsid protein contains nuclear import and export signals. Virol. J..

[114] Jacobs S.C., Taylor A., Herrero L.J., Mahalingam S., Fazakerley J.K. (2017). Mutation of a conserved nuclear export sequence in chikungunya virus capsid protein disrupts host cell nuclear import. Viruses.

[115] Taylor A., Liu X., Zaid A., Goh L.Y., Hobson-Peters J., Hall R.A., Merits A., Mahalingam S. (2017). Mutation of the N-Terminal Region of Chikungunya Virus Capsid Protein: Implications for Vaccine Design. MBio.

[116] Snyder A.J., Mukhopadhyay S. (2012). The Alphavirus E3 Glycoprotein Functions in a Clade-Specific Manner. J. Virol..

[117] Metz S.W., Geertsema C., Martina B.E., Andrade P., Heldens J.G., van Oers M.M., Vlak J.M., Pijlman G.P. (2011). Functional processing and secretion of Chikungunya virus E1 and E2 glycoproteins in insect cells. Virol. J..

[118] Li L., Jose J., Xiang Y., Kuhn R.J., Rossmann M.G. (2010). Structural changes of envelope proteins during alphavirus fusion. Nature.

[119] Uchime O., Fields W., Kielian M. (2013). The Role of E3 in pH Protection during Alphavirus Assembly and Exit. J. Virol..

[120] Cho B., Jeon B.Y., Kim J., Noh J., Kim J., Park M., Park S. (2008). Expression and evaluation of Chikungunya virus E1 and E2 envelope proteins for serodiagnosis of chikungunya virus infection. Yonsei Med. J..

[121] Salvador B., Zhou Y., Michault A., Muench M.O., Simmons G. (2009). Characterization of Chikungunya pseudotyped viruses: Identification of refractory cell lines and demonstration of cellular tropism differences mediated by mutations in E1 glycoprotein. Virology.

[122] Hussain K.M., Ching R., Lee H., Ng M.M. (2016). Establishment of a Novel Primary Human Skeletal Myoblast Cellular Model for Chikungunya Virus Infection and Pathogenesis. Nat. Publ. Gr..

[123] Gay B., Bernard E., Solignat M., Chazal N., Devaux C., Briant L. (2012). PH-dependent entry of chikungunya virus into Aedes albopictus cells. Infect. Genet. Evol..

[124] Wintachai P., Wikan N., Kuadkitkan A., Jaimipuk T., Ubol S., Pulmanausahakul R., Auewarakul P., Kasinrerk W., Weng W., Panyasrivanit M. (2012). Identification of Prohibitin as a Chikungunya Virus Receptor Protein. J. Med. Virol..

[125] Wintachai P., Thuaud F., Basmadjian C., Roytrakul S., Ubol S., Désaubry L., Smith D.R. (2015). Assessment of flavaglines as potential chikungunya virus entry inhibitors. Microbiol. Immunol..

[126] Thuaud F., Ribeiro N., Nebigil C.G., Désaubry L. (2013). Prohibitin ligands in cell death and survival: Mode of action and therapeutic potential. Chem. Biol..

[127] Fongsaran C., Jirakanwisal K., Kuadkitkan A., Wikan N., Wintachai P., Thepparit C., Ubol S., Phaonakrop N., Roytrakul S., Smith D.R. (2014). Involvement of ATP synthase β subunit in chikungunya virus entry into insect cells. Arch. Virol..

[128] Dudha N., Rana J., Rajasekharan S., Gabrani R., Gupta A., Chaudhary V.K., Gupta S. (2015). Host–pathogen interactome analysis of Chikungunya virus envelope proteins E1 and E2. Virus Genes.

[129] Jemielity S., Wang J.J., Chan Y.K., Ahmed A.A., Li W., Monahan S., Bu X., Farzan M., Freeman G.J., Umetsu D.T. (2013). TIM-family Proteins Promote Infection of Multiple Enveloped Viruses through Virion-associated Phosphatidylserine. PLoS Pathog..

[130] Mercer J., Helenius A. (2008). Vaccinia virus uses macropinocytosis and apoptotic mimicry to enter host cells. Science.

[131] Soares M.M., King S.W., Thorpe P.E. (2009). Targeting Inside-Out Phosphatidylserine as a Therapeutic Strategy For Viral Diseases. Nat. Med..

[132] Weber C., Berberich E., von Rhein C., Henß L., Hildt E., Schnierle B.S. (2017). Identification of Functional Determinants in the Chikungunya Virus E2 Protein. PLoS Negl. Trop. Dis..

[133] Ramsey J., Mukhopadhyay S. (2017). Disentangling the frames, the state of research on the alphavirus 6K and TF proteins. Viruses.

[134] Sanz M.A., Madan V., Carrasco L., Nieva J.L. (2003). Interfacial domains in sindbis virus 6K protein: Detection and functional characterization. J. Biol. Chem..

[135] Loewy A., Smyth J., von Bonsdorff C.H., Liljeström P., Schlesinger M.J. (1995). The 6-kilodalton membrane protein of Semliki Forest virus is involved in the budding process. J. Virol..

[136] Gaedigk-Nitschko K., Schlesinger M.J. (1990). The sindbis virus 6K protein can be detected in virions and is acylated with fatty acids. Virology.

[137] Lusa S., Garoff H., Liueström P. (1991). Fate of the 6K membrane protein of semliki forest virus during virus assembly. Virology.

[138] Taylor A., Melton J.V., Herrero L.J., Thaa B., Karo-Astover L., Gage P.W., Nelson M.A., Sheng K.-C., Lidbury B.A., Ewart G.D. (2016). Effects of an In-Frame Deletion of the *6k* Gene Locus from the Genome of Ross River Virus. J. Virol..

[139] Kuo S.C., Chen Y.J., Wang Y.M., Tsui P.Y., der Kuo M., Wu T.Y., Lo S.J. (2012). Cell-based analysis of Chikungunya virus E1 protein in membrane fusion. J. Biomed. Sci..

[140] Tsetsarkin K.A., Vanlandingham D.L., McGee C.E., Higgs S. (2007). A single mutation in Chikungunya virus affects vector specificity and epidemic potential. PLoS Pathog..

[141] Agarwal A., Sharma A.K., Sukumaran D., Parida M., Dash P.K. (2016). Two novel epistatic mutations (E1:K211E and E2:V264A) in structural proteins of Chikungunya virus enhance fitness in *Aedes aegypti*. Virology.

[142] Jones P.H., Maric M., Madison M.N., Maury W., Roller R.J., Okeoma C.M. (2013). BST-2/tetherin-mediated restriction of chikungunya (CHIKV) VLP budding is counteracted by CHIKV non-structural protein 1 (nsP1). Virology.

[143] Ooi Y.S., Dubé M., Kielian M. (2015). BST2/Tetherin inhibition of alphavirus exit. Viruses.

[144] Rupp J.C., Sokoloski K.J., Gebhart N.N., Hardy R.W. (2015). Alphavirus RNA synthesis and non-structural protein functions. J. Gen. Virol..

[145] Ahola T., Kaariainen L. (1995). Reaction in alphavirus mRNA capping: Formation of a covalent complex of nonstructural protein nsPl with 7-methyl-GMP. Proc. Natl. Acad. Sci. USA.

[146] Decroly E., Ferron F., Lescar J., Canard B. (2012). Conventional and unconventional mechanisms for capping viral mRNA. Nat. Rev. Microbiol..

[147] Ahola T., Merits A., Okeoma C.M. (2016). Functions of Chikungunya Virus Nonstructural Proteins. Chikungunya Virus Advances in Biology, Pathogenesis, and Treatment.

[148] Ahola T., Karlin D.G. (2015). Sequence analysis reveals a conserved extension in the capping enzyme of the alphavirus supergroup, and a homologous domain in nodaviruses. Biol. Direct..

[149] Laakkonen P., Ahola T., Kääriäinen L. (1996). The effects of palmitoylation on membrane association of Semliki Forest virus RNA capping enzyme. J. Biol. Chem..

[150] Lampio A., Kilpeläinen I., Pesonen S., Karhi K., Auvinen P., Somerharju P., Kääriäinen L. (2000). Membrane binding mechanism of an RNA virus-capping enzyme. J. Biol. Chem..

[151] Ahola T., Lampio A., Auvinen P., Kääriäinen L. (1999). Semliki Forest virus mRNA capping enzyme requires association with anionic membrane phospholipids for activity. EMBO J..

[152] Spuul P., Salonen A., Merits A., Jokitalo E., Kaariainen L., Ahola T. (2007). Role of the Amphipathic Peptide of Semliki Forest Virus Replicase Protein nsP1 in Membrane Association and Virus Replication. J. Virol..

[153] Fata C.L., Sawicki S.G., Sawicki D.L. (2002). Modification of Asn374 of nsP1 suppresses a Sindbis virus nsP4 minus-strand polymerase mutant. J. Virol..

[154] Shirako Y., Strauss E.G., Strauss J.H. (2000). Suppressor mutations that allow Sindbis virus RNA polymerase to function with nonaromatic amino acids at the N-terminus: Evidence for interaction between nsP1 and nsP4 in minus-strand RNA synthesis. Virology.

[155] Žusinaite E., Tints K., Kiiver K., Spuul P., Karo-Astover L., Merits A., Sarand I. (2007). Mutations at the palmitoylation site of non-structural protein nsP1 of Semliki Forest virus attenuate virus replication and cause accumulation of compensatory mutations. J. Gen. Virol..

[156] Mahauad-Fernandez W.D., Jones P.H., Okeoma C.M. (2014). Critical role for bone marrow stromal antigen 2 in acute Chikungunya virus infection. J. Gen. Virol..

[157] Russo A.T., White M.A., Watowich S.J. (2006). The Crystal Structure of the Venezuelan Equine Encephalitis Alphavirus nsP2 Protease. Structure.

[158] Shin G., Yost S.A., Miller M.T., Elrod E.J., Grakoui A., Marcotrigiano J. (2012). Structural and functional insights into alphavirus polyprotein processing and pathogenesis. Proc. Natl. Acad. Sci. USA.

[159] Nguyen P.T.V., Yu H., Keller P.A. (2015). Identification of chikungunya virus nsP2 protease inhibitors using structure-base approaches. J. Mol. Graph. Model..

[160] Das P.K., Merits A., Lulla A. (2014). Functional cross-talk between distant domains of chikungunya virus non-structural protein 2 is decisive for its RNA-modulating activity. J. Biol. Chem..

[161] Karpe Y.A., Aher P.P., Lole K.S. (2011). NTPase and 5′-RNA triphosphatase activities of chikungunya virus nsP2 protein. PLoS ONE.

[162] Ramakrishnan C., Kutumbarao N.H.V., Suhitha S., Velmurugan D. (2017). Structure–function relationship of Chikungunya nsP2 protease: A comparative study with papain. Chem. Biol. Drug Des..

[163] Vasiljeva L., Valmu L., Kääriäinen L., Merits A. (2001). Site-specific Protease Activity of the Carboxyl-terminal Domain of Semliki Forest Virus Replicase Protein nsP2. J. Biol. Chem..

[164] Peränen J., Rikkonen M., Liljeström P., Kääriäinen L. (1990). Nuclear localization of Semliki Forest virus-specific nonstructural protein nsP2. J. Virol..

[165] Fros J.J., Liu W.J., Prow N.A., Geertsema C., Ligtenberg M., Vanlandingham D.L., Schnettler E., Vlak J.M., Suhrbier A., Khromykh A.A. (2010). Chikungunya Virus Nonstructural Protein 2 Inhibits Type I/II Interferon-Stimulated JAK-STAT Signaling. J. Virol..

[166] Frolova E.I., Fayzulin R.Z., Cook S.H., Diane E., Rice C.M., Frolov I., Griffin D.E. (2002). Roles of Nonstructural Protein nsP2 and α/β Interferons in Determining the Outcome of Sindbis Virus Infection Roles of Nonstructural Protein nsP2 and α/β Interferons in Determining the Outcome of Sindbis Virus Infection. J. Virol..

[167] Breakwell L., Dosenovic P., Hedestam G.B.K., D’Amato M., Liljestrom P., Fazakerley J., McInerney G.M. (2007). Semliki Forest Virus Nonstructural Protein 2 Is Involved in Suppression of the Type I Interferon Response. J. Virol..

[168] Fros J.J., Major L.D., Scholte F.E.M., Gardner J., van Hemert M.J., Suhrbier A., Pijlman G.P. (2015). Chikungunya virus non-structural protein 2-mediated host shut-off disables the unfolded protein response. J. Gen. Virol..

[169] Akhrymuk I., Kulemzin S.V., Frolova E.I. (2012). Evasion of the Innate Immune Response: The Old World Alphavirus nsP2 Protein Induces Rapid Degradation of Rpb1, a Catalytic Subunit of RNA Polymerase II. J. Virol..

[170] Atasheva S., Gorchakov R., English R., Frolov I., Frolova E. (2007). Development of Sindbis Viruses Encoding nsP2/GFP Chimeric Proteins and Their Application for Studying nsP2 Functioning. J. Virol..

[171] Montgomery S.A., Berglund P., Beard C.W., Johnston R.E. (2006). Ribosomal Protein S6 Associates with Alphavirus Nonstructural Protein 2 and Mediates Expression from Alphavirus Messages. J. Virol..

[172] Mathur K., Anand A., Dubey S.K., Sanan-Mishra N., Bhatnagar R.K., Sunil S. (2016). Analysis of chikungunya virus proteins reveals that non-structural proteins nsP2 and nsP3 exhibit RNA interference (RNAi) suppressor activity. Sci. Rep..

[173] Rana J., Rajasekharan S., Gulati S., Dudha N., Gupta A., Chaudhary V.K., Gupta S. (2014). Network mapping among the functional domains of Chikungunya virus nonstructural proteins. Proteins Struct. Funct. Bioinform..

[174] Sreejith R., Rana J., Dudha N., Kumar K., Gabrani R., Sharma S.K., Gupta A., Vrati S., Chaudhary V.K., Gupta S. (2012). Mapping interactions of Chikungunya virus nonstructural proteins. Virus Res..

[175] Bourai M., Lucas-Hourani M., Gad H.H., Drosten C., Jacob Y., Tafforeau L., Cassonnet P., Jones L.M., Judith D., Couderc T. (2012). Mapping of Chikungunya Virus Interactions with Host Proteins Identified nsP2 as a Highly Connected Viral Component. J. Virol..

[176] Krejbich-Trotot P., Gay B., Li-Pat-Yuen G., Hoarau J.J., Jaffar-Bandjee M.C., Briant L., Gasque P., Denizot M. (2011). Chikungunya triggers an autophagic process which promotes viral replication. Virol. J..

[177] Judith D., Mostowy S., Bourai M., Gangneux N., Lelek M., Lucas-Hourani M., Cayet N., Jacob Y., Prévost M.C., Pierre P. (2013). Species-specific impact of the autophagy machinery on Chikungunya virus infection. EMBO Rep..

[178] Bakar F.A., Ng L. (2018). Nonstructural Proteins of Alphavirus—Potential Targets for Drug Development. Viruses.

[179] Malet H., Coutard B., Jamal S., Dutartre H., Papageorgiou N., Neuvonen M., Ahola T., Forrester N., Gould E.A., Lafitte D. (2009). The Crystal Structures of Chikungunya and Venezuelan Equine Encephalitis Virus nsP3 Macro Domains Define a Conserved Adenosine Binding Pocket. J. Virol..

[180] Vyas S., Matic I., Uchima L., Rood J., Zaja R., Hay R.T., Ahel I., Chang P. (2014). Family-wide analysis of poly(ADP-ribose) polymerase activity. Nat. Commun..

[181] Daniels C.M., Ong S.E., Leung A.K.L. (2015). The Promise of Proteomics for the Study of ADP-Ribosylation. Mol. Cell.

[182] McPherson R.L., Abraham R., Sreekumar E., Ong S.-E., Cheng S.-J., Baxter V.K., Kistemaker H.A.V., Filippov D.V., Griffin D.E., Leung A.K.L. (2017). ADP-ribosylhydrolase activity of Chikungunya virus macrodomain is critical for virus replication and virulence. Proc. Natl. Acad. Sci. USA.

[183] Lastarza M.W., Grakoui A., Rice C.M. (1994). Deletion and duplication mutations in the C-terminal nonconserved region of Sindbis virus nsP3: Effects on phosphorylation and on virus replication in vertebrate and invertebrate cells. Virology.

[184] Dé I., Fata-Hartley C., Sawicki S.G., Sawicki D.L. (2003). Functional Analysis of nsP3 Phosphoprotein Mutants of Sindbis Virus Functional Analysis of nsP3 Phosphoprotein Mutants of Sindbis Virus. J. Virol..

[185] Tuittila M., Hinkkanen A.E. (2003). Amino acid mutations in the replicase protein nsP3 of Semliki Forest virus cumulatively affect neurovirulence. J. Gen. Virol..

[186] Neuvonen M., Kazlauskas A., Martikainen M., Hinkkanen A., Ahola T., Saksela K. (2011). SH3 domain-mediated recruitment of host cell amphiphysins by alphavirus nsp3 promotes viral RNA replication. PLoS Pathog..

[187] Tossavainen H., Aitio O., Hellman M., Saksela K., Permi P. (2016). Structural basis of the high affinity interaction between the Alphavirus nonstructural protein-3 (nsP3) and the SH3 domain of amphiphysin-2. J. Biol. Chem..

[188] Mim C., Unger V.M. (2012). Membrane curvature and its generation by BAR proteins. Trends Biochem. Sci..

[189] Fros J.J., Domeradzka N.E., Baggen J., Geertsema C., Flipse J., Vlak J.M., Pijlman G.P. (2012). Chikungunya Virus nsP3 Blocks Stress Granule Assembly by Recruitment of G3BP into Cytoplasmic Foci. J. Virol..

[190] Panas M.D., Ahola T., McInerney G.M. (2014). The C-Terminal Repeat Domains of nsP3 from the Old World Alphaviruses Bind Directly to G3BP. J. Virol..

[191] Panas M.D., Varjak M., Lulla A., Eng K.E., Merits A., Hedestam G.B.K., McInerney G.M. (2012). Sequestration of G3BP coupled with efficient translation inhibits stress granules in Semliki Forest virus infection. Mol. Biol. Cell.

[192] Scholte F.E.M., Tas A., Albulescu I.C., Žusinaite E., Merits A., Snijder E.J., van Hemert M.J. (2015). Stress Granule Components G3BP1 and G3BP2 Play a Proviral Role Early in Chikungunya Virus Replication. J. Virol..

[193] Kim D.Y., Reynaud J.M., Rasalouskaya A., Akhrymuk I., Mobley J.A., Frolov I., Frolova E.I. (2016). New World and Old World Alphaviruses Have Evolved to Exploit Different Components of Stress Granules, FXR and G3BP Proteins, for Assembly of Viral Replication Complexes. PLoS Pathog..

[194] Schulte T., Liu L., Panas M.D., Thaa B., Dickson N., Götte B., Achour A., Mcinerney G.M. (2016). Combined structural, biochemical and cellular evidence demonstrates that both FGDF motifs in alphavirus nsP3 are required for efficient replication. Open Biol..

[195] Fros J.J., Geertsema C., Zouache K., Baggen J., Domeradzka N., van Leeuwen D.M., Flipse J., Vlak J.M., Failloux A.B., Pijlman G.P. (2015). Mosquito Rasputin interacts with chikungunya virus nsP3 and determines the infection rate in Aedes albopictus. Parasites Vectors.

[196] Remenyi R., Roberts G.C., Zothner C., Merits A., Harris M. (2017). SNAP-Tagged Chikungunya Virus Replicons Improve Visualisation of Non-Structural Protein 3 by Fluorescence Microscopy. Sci. Rep..

[197] Reid S.P., Tritsch S.R., Kota K., Chiang C.-Y., Dong L., Kenny T., Brueggemann E.E., Ward M.D., Cazares L.H., Bavari S. (2015). Sphingosine kinase 2 is a chikungunya virus host factor co-localized with the viral replication complex. Emerg. Microbes Infect..

[198] Lark T., Keck F., Narayanan A. (2018). Interactions of Alphavirus nsP3 protein with host proteins. Front. Microbiol..

[199] Rathore A.P.S., Haystead T., Das P.K., Merits A., Ng M.L., Vasudevan S.G. (2014). Chikungunya virus nsP3 & nsP4 interacts with HSP-90 to promote virus replication: HSP-90 inhibitors reduce CHIKV infection and inflammation in vivo. Antiviral Res..

[200] Rupp J.C., Jundt N., Hardy R.W. (2011). Requirement for the Amino-Terminal Domain of Sindbis Virus nsP4 during Virus Infection. J. Virol..

[201] Chen M.W., Tan Y.B., Zheng J., Zhao Y., Lim B.T., Cornvik T., Lescar J., Ng L.F.P., Luo D. (2017). Chikungunya virus nsP4 RNA-dependent RNA polymerase core domain displays detergent-sensitive primer extension and terminal adenylyltransferase activities. Antiviral Res..

[202] Rathore A.P.S., Ng M.L., Vasudevan S.G. (2013). Differential unfolded protein response during Chikungunya and Sindbis virus infection: CHIKV nsP4 suppresses eIF2a phosphorylation. Virol. J..

[203] Pietilä M.K., Hellström K., Ahola T. (2017). Alphavirus polymerase and RNA replication. Virus Res..

[204] Paingankar M.S., Arankalle V.A. (2014). Identification of chikungunya virus interacting proteins in mammalian cells. J. Biosci..

[205] Treffers E.E., Tas A., Scholte F.E.M., Van M.N., Heemskerk M.T., de Ru A.H., Snijder E.J., van Hemert M.J., van Veelen P.A. (2015). Temporal SILAC-based quantitative proteomics identifies host factors involved in chikungunya virus replication. Proteomics.

[206] Karlas A., Berre S., Couderc T., Varjak M., Braun P., Meyer M., Gangneux N., Karo-Astover L., Weege F., Raftery M. (2016). A human genome-wide loss-of-function screen identifies effective chikungunya antiviral drugs. Nat. Commun..

[207] Gutierrez F.J., Perlman S., Sanchez-aparicio M.T., Garcı A., Enjuanes L., Sola I. (2018). MERS-CoV 4b protein interferes with the NF-κB-dependent innate immune response during infection. PLoS Pathog..

[208] Farjot G., Sergeant A., Mikaelian I. (1999). A novel nucleoporin-like protein interacts with both HIV-1 Rev nuclear export signal and CRM-1. J. Biol. Chem..

[209] Elton D., Simpson-holley M., Archer K., Hallam R., Mccauley J., Digard P., Medcalf L.I.Z., Cauley J.M.C. (2001). Interaction of the Influenza Virus Nucleoprotein with the Cellular CRM1-Mediated Nuclear Export Pathway. J. Virol..

[210] Hakata Y., Yamada M., Mabuchi N., Shida H. (2002). The carboxy-terminal region of the human immunodeficiency virus type 1 protein Rev has multiple roles in mediating CRM1-related Rev functions. J. Virol..

[211] Hakata Y., Yamada M., Shida H. (2003). A multifunctional domain in human CRM1 (exportin 1) mediates RanBP3 binding and multimerization of human T-cell leukemia virus type 1 Rex protein. Mol. Cell. Biol..

[212] Yedavalli V.S.R.K., Neuveut C., Chi Y.H., Kleiman L., Jeang K.T. (2004). Requirement of DDX3 DEAD box RNA helicase for HIV-1 Rev-RRE export function. Cell.

[213] Daelemans D., Costes S.V., Lockett S., Pavlakis G.N. (2005). Kinetic and Molecular Analysis of Nuclear Export Factor CRM1 Association with Its Cargo In Vivo Kinetic and Molecular Analysis of Nuclear Export Factor CRM1 Association with Its Cargo In Vivo. Technology.

[214] Hardes K., Becker G.L., Lu Y., Dahms S.O., Köhler S., Beyer W., Sandvig K., Yamamoto H., Lindberg I., Walz L. (2015). Novel Furin Inhibitors with Potent Anti-infectious Activity. ChemMedChem.

[215] Kuadkitkan A., Wikan N., Fongsaran C., Smith D.R. (2010). Identification and characterization of prohibitin as a receptor protein mediating DENV-2 entry into insect cells. Virology.

[216] Liu S., Wang W., Brown L.E., Qiu C., Lajkiewicz N., Zhao T., Zhou J., Porco J.A., Wang T.T. (2015). A Novel Class of Small Molecule Compounds that Inhibit Hepatitis C Virus Infection by Targeting the Prohibitin-CRaf Pathway. EBioMedicine.

[217] Emerson V., Holtkotte D., Pfeiffer T., Wang I.-H., Schnolzer M., Kempf T., Bosch V. (2010). Identification of the Cellular Prohibitin 1/Prohibitin 2 Heterodimer as an Interaction Partner of the C-Terminal Cytoplasmic Domain of the HIV-1 Glycoprotein. J. Virol..

[218] Brenndorfer E.D., Brass A., Karthe J., Ahlen G., Bode J.G., Sallberg M. (2014). Cleavage of the T Cell Protein Tyrosine Phosphatase by the Hepatitis C Virus Nonstructural 3/4A Protease Induces a Th1 to Th2 Shift Reversible by Ribavirin Therapy. J. Immunol..

[219] Thach D.C., Stenger D.A. (2003). Effects of collagen matrix on Sindbis virus infection of BHK cells. J. Virol. Methods.

[220] Adams L.D., Tomasselli A.G., Robbins P., Moss B., Heinrikson R.L. (1992). HIV-1 protease cleaves actin during acute infection of human T-lymphocytes. AIDS Res. Hum. Retrovir..

[221] Feldman S.A., Audet S., Beeler J.A. (2000). The Fusion Glycoprotein of Human Respiratory Syncytial Virus Facilitates Virus Attachment and Infectivity via an Interaction with Cellular Heparan Sulfate. J. Virol..

[222] Silberstein E., Dveksler G., Kaplan G.G. (2001). Neutralization of hepatitis A virus (HAV) by an immunoadhesin containing the cysteine-rich region of HAV cellular receptor-1. J. Virol..

[223] Milewska A., Zarebski M., Nowak P., Stozek K., Potempa J., Pyrc K. (2014). Human Coronavirus NL63 Utilizes Heparan Sulfate Proteoglycans for Attachment to Target Cells. J. Virol..

[224] Leistner C.M., Gruen-Bernhard S., Glebe D. (2008). Role of glycosaminoglycans for binding and infection of hepatitis B virus. Cell. Microbiol..

[225] Galloway D.A., Laimins L.A., Division B., Hutchinson F. (2016). Glycosaminoglycans and infection. Front. Biosci..

[226] Kondratowicz A.S., Lennemann N.J., Sinn P.L., Davey R.A., Hunt C.L., Moller-Tank S., Meyerholz D.K., Rennert P., Mullins R.F., Brindley M. (2011). T-cell immunoglobulin and mucin domain 1 (TIM-1) is a receptor for Zaire Ebolavirus and Lake Victoria Marburgvirus. Proc. Natl. Acad. Sci. USA.

[227] Kaplan G., Totsuka A., Thompson P., Akatsuka T., Moritsugu Y., Feinstonel S.M., Feinstone S.M. (1996). Identification of a surface glycoprotein on African green monkey kidney cells as a receptor for hepatitis A virus. EMBO J..

[228] Feigelstock D., Thompson P., Mattoo P., Zhang Y., Kaplan G.G. (1998). The human homolog of HAVcr-1 codes for a hepatitis A virus cellular receptor. J. Virol..

[229] Shimojima M., Takada A., Ebihara H., Neumann G., Fujioka K., Irimura T., Jones S., Feldmann H., Kawaoka Y. (2006). Tyro3 Family-Mediated Cell Entry of Ebola and Marburg Viruses. J. Virol..

[230] Shimojima M., Stroher U., Ebihara H., Feldmann H., Kawaoka Y. (2012). Identification of Cell Surface Molecules Involved in Dystroglycan-Independent Lassa Virus Cell Entry. J. Virol..

[231] Morizono K., Xie Y., Olafsen T., Lee B., Dasgupta A., Wu A.M., Chen I.S.Y. (2011). The Soluble serum protein gas6 bridges virion envelope phosphatidylserine to the TAM receptor tyrosine kinase Axl to mediate viral entry. Cell Host Microbe.

[232] Burstein E., Hoberg J.E., Wilkinson A.S., Rumble J.M., Csomos R.A., Komarck C.M., Maine G.N., Wilkinson J.C., Mayo M.W., Duckett C.S. (2005). COMMD proteins, a novel family of structural and functional homologs of MURR1. J. Biol. Chem..

[233] Taura M., Kudo E., Kariya R., Goto H., Matsuda K., Hattori S., Vaeteewoottacharn K., McDonald F., Suico M.A., Shuto T. (2015). COMMD1/Murr1 Reinforces HIV-1 Latent Infection through IκB-α Stabilization. J. Virol..

[234] Presser L.D., Haskett A., Waris G. (2011). Hepatitis C virus-induced furin and thrombospondin-1 activate TGF-β1: Role of TGF-β1 in HCV replication. Virology.

[235] Greber U.F., Way M. (2006). A superhighway to virus infection. Cell.

[236] Lukic Z., Dharan A., Fricke T., Diaz-Griffero F., Campbell E.M. (2014). HIV-1 Uncoating Is Facilitated by Dynein and Kinesin 1. J. Virol..

[237] Watanabe R., Leser G.P., Lamb R.A. (2011). Influenza virus is not restricted by tetherin whereas influenza VLP production is restricted by tetherin. Virology.

[238] Dafa-Berger A., Kuzmina A., Fassler M., Yitzhak-Asraf H., Shemer-Avni Y., Taube R. (2012). Modulation of hepatitis C virus release by the interferon-induced protein BST-2/tetherin. Virology.

[239] Kaletsky R.L., Francica J.R., Agrawal-Gamse C., Bates P. (2009). Tetherin-mediated restriction of filovirus budding is antagonized by the Ebola glycoprotein. Proc. Natl. Acad. Sci. USA.

[240] Radoshitzky S.R., Dong L., Chi X., Clester J.C., Retterer C., Spurgers K., Kuhn J.H., Sandwick S., Ruthel G., Kota K. (2010). Infectious Lassa Virus, but Not Filoviruses, Is Restricted by BST-2/Tetherin. J. Virol..

[241] Weidner J.M., Jiang D., Pan X.-B., Chang J., Block T.M., Guo J.-T. (2010). Interferon-Induced Cell Membrane Proteins, IFITM3 and Tetherin, Inhibit Vesicular Stomatitis Virus Infection via Distinct Mechanisms. J. Virol..

[242] Pardieu C., Vigan R., Wilson S.J., Calvi A., Zang T., Bieniasz P., Kellam P., Towers G.J., Neil S.J.D. (2010). The RING-CH Ligase K5 Antagonizes Restriction of KSHV and HIV-1 Particle Release by Mediating Ubiquitin-Dependent Endosomal Degradation of Tetherin. PLoS Pathog..

[243] Neil S.J.D., Zang T., Bieniasz P.D. (2008). Tetherin inhibits retrovirus release and is antagonized by HIV-1 Vpu. Nature.

[244] Perez-Caballero D., Zang T., Ebrahimi A., McNatt M.W., Gregory D.A., Johnson M.C., Bieniasz P.D. (2009). Tetherin Inhibits HIV-1 Release by Directly Tethering Virions to Cells. Cell.

[245] Fitzgerald K.D., Chase A.J., Cathcart A.L., Tran G.P., Semler B.L. (2013). Viral Proteinase Requirements for the Nucleocytoplasmic Relocalization of Cellular Splicing Factor SRp20 during Picornavirus Infections. J. Virol..

[246] Issac T.H.K., Tan E.L., Chu J.J.H. (2014). Proteomic profiling of chikungunya virus-infected human muscle cells: Reveal the role of cytoskeleton network in CHIKV replication. J. Proteomics.

[247] Wu W., Panté N. (2016). Vimentin plays a role in the release of the influenza A viral genome from endosomes. Virology.

[248] Conwell S.E., White A.E., Harper J.W., Knipe D.M. (2015). Identification of TRIM27 as a Novel Degradation Target of Herpes Simplex Virus 1 ICP0. J. Virol..

[249] Oakland T.E., Haselton K.J., Randall G. (2013). EWSR1 Binds the Hepatitis C Virus cis-Acting Replication Element and Is Required for Efficient Viral Replication. J. Virol..

[250] Leung J.K., Berube N., Venable S., Ahmed S., Timchenko N., Pereira-Smith O.M. (2001). MRG15 Activates the B-myb Promoter through Formation of a Nuclear Complex with the Retinoblastoma Protein and the Novel Protein PAM14. J. Biol. Chem..

[251] Poenisch M., Metz P., Blankenburg H., Ruggieri A., Lee J.Y., Rupp D., Rebhan I., Diederich K., Kaderali L., Domingues F.S. (2015). Identification of HNRNPK as Regulator of Hepatitis C Virus Particle Production. PLoS Pathog..

[252] Dinh P.X., Das A., Franco R., Pattnaik A.K. (2013). Heterogeneous Nuclear Ribonucleoprotein K Supports Vesicular Stomatitis Virus Replication by Regulating Cell Survival and Cellular Gene Expression. J. Virol..

[253] Nakatsu F., Baskin J.M., Chung J., Tanner L.B., Shui G., Lee S.Y., Pirruccello M., Hao M., Ingolia N.T., Wenk M.R. (2012). Its Impact on Plasma Membrane Identity. J. Cell Biol..

[254] Zhang J., Sze D.M.Y., Yung B.Y.M., Tang P., Chen W.J., Chan K.H., Leung P.H.M. (2016). Distinct expression of interferon-induced protein with tetratricopeptide repeats (IFIT) 1/2/3 and other antiviral genes between subsets of dendritic cells induced by dengue virus 2 infection. Immunology.

[255] Woznik M., Rödner C., Lemon K., Rima B., Mankertz A., Finsterbusch T. (2010). Mumps virus small hydrophobic protein targets ataxin-1 ubiquitin-like interacting protein (ubiquilin 4). J. Gen. Virol..

[256] Han Z., Sagum C.A., Takizawa F., Ruthel G., Berry C.T., Kong J., Sunyer J.O., Freedman B.D., Bedford M.T., Sidhu S.S. (2017). Ubiquitin Ligase WWP1 Interacts with Ebola Virus VP40 to Regulate Egress. J. Virol..

[257] Ma-Lauer Y., Carbajo-Lozoya J., Hein M.Y., Müller M.A., Deng W., Lei J., Meyer B., Kusov Y., von Brunn B., Bairad D.R. (2016). p53 down-regulates SARS coronavirus replication and is targeted by the SARS-unique domain and PL^pro^ via E3 ubiquitin ligase RCHY1. Proc. Natl. Acad. Sci. USA.

[258] Bangs P., Burke B., Powers C., Craig R., Purohit A., Doxsey S. (1998). Functional analysis of Tpr: Identification of nuclear pore complex association and nuclear localization domains and a role in mRNA export. J. Cell Biol..

[259] Duprex W.P., McQuaid S., Rima B.K. (2000). Measles virus-induced disruption of the glial-fibrillary-acidic protein cytoskeleton in an astrocytoma cell line (U-251). J. Virol..

[260] Huang W.R., Chiu H.C., Liao T.L., Chuang K.P., Shih W.L., Liu H.J. (2015). Avian reovirus protein p17 functions as a nucleoporin Tpr suppressor leading to activation of p53, p21 and PTEN and inactivation of PI3K/AKT/mTOR and ERK signaling pathways. PLoS ONE.

[261] Leymarie O., Meyer L., Tafforeau L., Lotteau V., da Costa B., Delmas B., Chevalier C., le Goffic R. (2017). Influenza virus protein PB1-F2 interacts with CALCOCO2 (NDP52) to modulate innate immune response. J. Gen. Virol..

[262] Münz C. (2013). Macroautophagy—Friend or foe of viral replication?. EMBO Rep..

[263] Mannova P., Beretta L., Mannová P. (2005). Activation of the N-Ras–PI3K–Akt–mTOR Pathway by Hepatitis C Virus: Control of Cell Survival and Viral Replication. J. Virol..

[264] Soares J.A.P., Leite F.G.G., Andrade L.G., Torres A.A., de Sousa L.P., Barcelos L.S., Teixeira M.M., Ferreira P.C.P., Kroon E.G., Souto-Padron T. (2009). Activation of the PI3K/Akt Pathway Early during Vaccinia and Cowpox Virus Infections Is Required for both Host Survival and Viral Replication. J. Virol..

[265] Jiménez V.C., Martinez F.O., Booiman T., van Dort K.A., van de Klundert M.A.A., Gordon S., Geijtenbeek T.B.H., Kootstra N.A. (2015). G3BP1 restricts HIV-1 replication in macrophages and T-cells by sequestering viral RNA. Virology.

[266] Bidet K., Dadlani D., Garcia-Blanco M.A. (2014). G3BP1, G3BP2 and CAPRIN1 Are Required for Translation of Interferon Stimulated mRNAs and Are Targeted by a Dengue Virus Non-coding RNA. PLoS Pathog..

[267] Wang R.Y.L., Kuo R.L., Ma W.C., Huang H.I., Yu J.S., Yen S.M., Huang C.R., Shih S.R. (2013). Heat shock protein-90-beta facilitates enterovirus 71 viral particles assembly. Virology.

[268] Das I., Basantray I., Mamidi P., Nayak T.K., Pratheek B.M., Chattopadhyay S., Chattopadhyay S. (2014). Heat shock protein 90 positively regulates Chikungunya virus replication by stabilizing viral non-structural protein nsP2 during infection. PLoS ONE.

[269] Wang Y., Li Y., Ding T. (2017). Heat shock protein 90β in the Vero cell membrane binds Japanese encephalitis virus. Int. J. Mol. Med..

[270] Liu D., Wu A., Cui L., Hao R., Wang Y., He J., Guo D. (2014). Hepatitis B virus polymerase suppresses NF-κB signaling by inhibiting the activity of IKKs via interaction with Hsp90β. PLoS ONE.

[271] Montero H., Rojas M., Arias C.F., López S. (2008). Rotavirus infection induces the phosphorylation of eIF2α but prevents the formation of stress granules. J. Virol..

[272] Wylie K.M., Schrimpf J.E., Morrison L.A. (2009). Increased eIF2α phosphorylation attenuates replication of herpes simplex virus 2 vhs mutants in mouse embryonic fibroblasts and correlates with reduced accumulation of the PKR antagonist ICP34.5. J. Virol..

